# Structural Equation Modeling Reveals How Allometry Shapes Integration in Avian Cranial Evolution

**DOI:** 10.1093/icb/icag106

**Published:** 2026-07-02

**Authors:** J W Oyston, J T Thorson, A Knapp, R D Marek, R N Felice

**Affiliations:** Division of Cell and Developmental Biology, Centre for Integrative Anatomy, University College London, Gower Street, WC1E 6BT, London, UK; Resource Ecology and Fisheries Management Division, Alaska Fisheries Science Center, 7600 Sand Point Way N.E., WA 98115, Seattle, USA; Division of Cell and Developmental Biology, Centre for Integrative Anatomy, University College London, Gower Street, WC1E 6BT, London, UK; Division of Cell and Developmental Biology, Centre for Integrative Anatomy, University College London, Gower Street, WC1E 6BT, London, UK; Division of Cell and Developmental Biology, Centre for Integrative Anatomy, University College London, Gower Street, WC1E 6BT, London, UK; Science Group, Natural History Museum, Cromwell Road, SW7 5BD, London, UK

## Abstract

Testing hypotheses of phenotypic modularity involves assessing whether groups of traits covary more strongly with each other than with parts outside the group. Structural equation modeling (SEM) is a flexible statistical framework for interrogating complex relationships between sets of variables, making it ideally suited to studies of hierarchical modularity and integration. However, quantifying the modular organization of high-dimensional traits using SEM in a phylogenic context has only recently become possible through new methodological advances. Here, we applied SEM to investigate patterns and correlates of phenotypic modularity in the skull and brain of birds. Birds independently evolved relatively large brains multiple times, as well as a wide range of different skull and brain morphologies. While some have proposed the bird skull is composed of several functional or developmental modules, others have suggested the skull is highly integrated, with allometric scaling structuring trait correlations. The data best supported a model in which brain shape is influenced by a hard tissue module consisting of neurocranium and rostrum (RS) shape, as well as the jaw musculature. RS itself does not strongly covary with other aspects of the skull and brain however, suggesting decoupling of beak morphology from the rest of the avian cranium. All variables, with the exception of RS, are strongly influenced by size, supporting the idea that allometry is a major influence on craniofacial integration in birds. These results provide new insights into likely drivers shaping the evolution of the skull in birds and highlight the usefulness of *phyloSEM* testing hypotheses of evolutionary modularity and integration.

## Introduction

Modularity is a property of complex structures or systems where covariation between parts within specific subsets (modules) is greater than the covariation between subsets, due to some common factor (Zelditch and Goswami 2021). This concept is closely related to integration, which describes the strength of covariation between parts (Olson and Miller 1958; Bookstein 2022). For morphological traits, modules are often defined based on the function, mechanics, composition or shared development of biological structures (Zelditch et al. 2008; Goswami et al. 2012; Felice and Goswami 2018; Evans et al. 2021). Because modularity and integration describe how variance is organized and because (heritable) variance is the raw material upon which natural selection can act, modularity patterns can help to characterize how traits are organized into units that can evolve. For example, if modules are, to some degree, functionally independent, selective forces could operate differently on them, allowing them to evolve independently. There is disagreement on whether modularity is a prerequisite for the evolvability of traits themselves and how strongly traits must be integrated with each other to provide “selectively useful variation” (Gerhart and Kirschner 2007).

Nonetheless, it is clear that understanding the patterns of interactions among traits is essential for characterizing the evolution of phenotypic variation through time. Much of the research on phenotypic and evolutionary modularity has focused on the possible role of developmental (Williams and Nagy 2001; Porto et al. 2009; Lacquaniti et al. 2013; Goswami et al. 2023) and functional (Goswami 2006; Goswami and Polly 2010; Blanke 2018; Dellinger et al. 2019; Burns et al. 2023) interactions in determining boundaries between hierarchically nested modules. For example, ontogeny has long been associated with cranial modularity and integration (Klingenberg 2013; Hallgrímsson et al. 2019). Changes in the timing of development (heterochrony) have been linked to evolutionary changes in cranial shape in both birds (Bhullar et al. 2012) and mammals (Kyomen et al. 2023). As individual skull regions have been shown to exhibit differing levels of integration at different stages of ontogeny (Arnold et al. 2025), heterochronic changes can influence patterns of modularity.

Despite the inferred importance of developmental timing and heterochrony on shaping integration and modularity, the influence of allometric scaling on the relationships between anatomical modules has received comparatively little attention (Vasseur et al. 2022). Research on this association has largely focused on “evolutionary allometry,” analysing covariation between variables in different lineages to determine which components of shape evolution can be attributed to allometry and which are influenced by other factors (Klingenberg and Marugán-Lobón 2013; Smaers et al. 2021; Zelditch and Swiderski 2023a). For example, in armadillos, allometric patterns were found to vary heterogeneously across different regions on the cranium as well as ontogenetically and across species (Le Verger et al. 2024), suggesting allometry and modularity may be closely linked. A strong link between allometry and modularity seems to be present in parrots, where almost half of the variation in skull and beak shape can be predicted by allometry and structural integration (Bright et al. 2019).

One reason why shared allometric scaling might generate strong integration is because trait covariation and developmental growth trajectories are both strongly influenced by the generative processes underlying the expression and growth of those traits (Hallgrímsson et al. 2019; Jablonski 2020; Vea and Shingleton 2021). These generative processes include gene regulatory networks (GRNs) or shared developmental pathways. However, directly measuring GRNs or developmental mechanisms in comparative evolutionary studies remains extremely challenging. Multivariate approaches that can model the complex hierarchical patterns of covariance relationships generated by these processes are therefore desirable. Additionally, variables used to describe biological variability rarely, if ever, represent complete descriptions of their nature. Although all models are abstractions designed to reduce the complexity of the system to the core elements of interest, there are cases where proxies capture different aspects of a variable in ways which could affect which covariance relationships are recovered and how strongly they are supported. Studies of allometry represent one such example, where the observed variables (e.g., masses, volumes, ratios) are all proxies of “size” which often show different scaling relationships with other variables.

There is therefore a need for statistical methods summarizing how these different proxies covary together and how that covariance itself correlates with other variables, to gain a more complete understanding of how allometry impacts modularity. Structural equation modeling (SEM) is a multivariate statistical framework (Fox et al. 2013; Harlow 2023) deriving from path analysis (Wright 1921; Voyer and Garamszegi 2014; Shipley 2016), designed to evaluate hypotheses of causal relationships between variables. Three features of SEMs make them well suited to hierarchical studies of modularity. First, variables in SEMs can be dependent variables in some equations in the model and predictor variables in others, including reciprocal interactions directly or through intermediate variables. Second, SEMs allow modeling of relationships with multiple dependent variables as well as multiple predictors. Third, a key feature of SEMs which distinguishes them from other multivariate methods (Clavel et al. 2015; Bartoszek et al. 2024) is the ability to include latent variables, unobserved constructs which are hypothesized predictors of covariation in observed dependent variables (Lawley and Maxwell 1962; Kline 2023). In geometric morphometric (GMM) studies, the landmark coordinates are the observed variables, while the latent variables are estimates of the underlying anatomical and developmental systems which explain covariation in shape. Unlike traditional PCA, SEM allows the relationships between observed and latent variables to be specified to test hypotheses regarding the underlying systems producing variation (David Polly et al. 2013; Greenacre et al. 2022).Traditional GMM methods are well suited to investigating multivariate relationships between a wide range of variable types, taking into account phylogeny. However, they are not typically able to investigate hierarchical levels of modularity directly (although see Tseng et al. 2025). SEMs, for the reasons given above, are well suited to investigating hierarchical covariance patterns among modules but have traditionally been computationally demanding and unable to account for phylogenetic covariance. Recent advances also allow SEMs to control for phylogenetic correlations between data from different lineages using a range of models (Thorson and van der Bijl 2023), allowing SEMs to now be applied to study correlated evolution in a wide range of contexts.

The avian head is an ideal system to investigate modularity. Phenotypic variation in the avian skull (Zusi 1993; Natale and Slater 2022) is thought to reflect adaptation and ecological specialization (Tokita et al. 2017; Hunt et al. 2023). Previous studies suggest that the avian cranium is highly modular, with particular regions evolving at different rates reflecting their developmental origin (Felice and Goswami 2018). As with any modular biological system (Klingenberg 2008), the modules that make up the avian skull are only *semi*-independent and nonetheless exhibit variational and evolutionary correlations (Marugán-Lobón and Nebreda 2025). For example, regions of the avian skull are thought to be integrated due to developmental or functional constraints (Klingenberg and Marugán-Lobón 2013; Bright et al. 2016; Hunt et al. 2023). Other aspects of bird skull shape have been shown to covary allometrically (Natale and Slater 2022; Korkmazcan et al. 2025), likely due to changes in developmental timing (Bhullar et al. 2016; Plateau and Foth 2020).

Along with beak and cranial shape, changes in brain size and shape have been proposed as key factors influencing craniofacial growth and evolution. Brain size is strongly correlated with eye size (Burton 2008) and both orbit and brain shape seem to covary to accommodate changes in overall skull shape (Kawabe et al. 2013). The skull roof also seems to track changes in brain shape in reptiles more generally, including birds, suggesting deep-rooted developmental links between the two structures (Fabbri et al. 2017; Watanabe et al. 2019).

Several interrelated hypotheses have been proposed to explain the proximate causes of these trait interactions. For example, the “Hand in Glove” hypothesis (Richtsmeier and Flaherty 2013), which proposes that the neurocranium evolves primarily to accommodate changes in brain size. However, increases in relative brain size appear to be correlated with major components of skull shape variation, specifically those related to basicranial flexion, and that these changes are independent of allometry (Marugán-Lobón et al. 2022). This would seem to support the “Spatial Packing” hypothesis, in which integrated shape changes in the skull and brain occur in response to maintaining functional integrity within the limited space in the head (Jeffery et al. 2021). Functional performance of the skull and beak in feeding is also dependent on the relative size and orientation of the jaw musculature (Van Der Meij and Bout 2008). The “Functional Matrix” hypothesis (Moss and Young 1960; Moss 1971) predicts that the importance of soft tissues relative to cranial shape itself cause the skull to evolve in response to the functional demands placed upon it by the associated muscular on the one hand and the brain on the other.

Although these proposed mechanisms of brain–skull correlation are well supported from developmental experiments of model organisms, there remain unanswered questions about the strength and patterns of evolutionary correlations among these traits. Here, we use *phyloSEM* and latent variables to interrogate these correlations. We used a two-stage approach to capture the hierarchical modular relationships in the brain and skull of birds. First, factor analysis (FA) was used to summarize the covariation between landmarks for each trait and region of the brain. Second, we tested the fit of SEMs representing different hypotheses of the groupings of these regions into models and the relationships between them. Our aims were to quantify the directionality of trait interactions (i.e., does shape evolution in the brain influence shape evolution in the skull or vice versa) to help to untangle how the aforementioned developmental interactions influence or bias evolutionary change. We also incorporated data about brain and body size scaling to test how allometric scaling influences patterns of evolutionary modularity and integration.

## Method

### Dataset compilation

We carried out shape analyses of morphology using the dataset of Knapp et al. (2025). Landmark data were collected for 322 CT scans of bird skulls representing 311 extant and 11 extinct species spanning the whole tree of crown birds. In total, the dataset represented 43 out of 44 orders, 188 of 254 families, and 322 of 2392 genera of extant birds (Gill et al. 2024). The landmarking scheme consists of 20 anatomical landmarks and 190 semilandmarks in 21 curves. Endocast shape data were also taken from the same dataset and consists of 14 anatomical landmarks, 72 semilandmarks in 15 curves, and 103 surface semilandmarks. Endocasts were generated from skull meshes using the “endomaker” function of the R package *Arothron* (Profico et al. 2021). The remnants of the cranial nerve had been manually removed from these scans to allow landmarking of the optic lobe. General Procrustes alignment (GPA) was carried out on all skull landmarks, all endocast landmarks as well as skull landmark subsets (rostrum, neurocranium, and jaw muscles) separately. These GMM datasets consist of landmarks and semilandmarks digitized only on the right side of the skull or cranial endocast. We thus reflected all right-side landmarks across the midline before GPA and removed the imputed landmarks for all subsequent analyses (Knapp et al. 2025). This approach removes undesirable alignment artifacts that can sometimes occur as part of GPA of only one side of bilaterally symmetrical structures (Zelditch et al. 2012). Some authors have argued that high-density morphometric data and the sliding of semilandmarks can bias inferences about variance and covariance (Cardini 2019). However, recent simulation studies have demonstrated that semilandmarks rarely mislead modularity and integration analyses (Zelditch and Swiderski 2023b) and may in fact improve the accuracy of such analyses, for example by reducing boundary bias (Goswami et al. 2019).

We also took body mass estimates from Knapp et al. (2025), based on the mean body mass taken from AVONET (Tobias et al. 2022) for extant species and either published or estimated values based on hindlimb scaling (Field et al. 2013) for extinct taxa. We took brain volume estimates from those compiled in Oyston et al. (2025), including all species in Knapp et al. (2025) . We logged both body mass and brain volume before analysis.

### Phylogeny

We based our analyses on a recent, well-supported molecular phylogeny with broad comprehensive coverage of living birds (Claramunt et al. 2024). First, we substituted extant taxa with a single congener which were not in the Claramunt et al. (2024) phylogeny for that congener if it was included in the phylogeny. Otherwise, we omitted them from the phylogeny and did not include them in analyses. Second, fossil taxa were grafted onto this backbone phylogeny based on a recent topology of Mesozoic birds (Wang and Lloyd 2016). Due to the Claramunt et. al. (2024) inferring an older age for crown birds than the Wang et al. tree (107.18 Ma rather than 103.2 Ma), we shortened the branch leading to the most recent common ancestor of *Ichthyornis* and crown birds by 5 Ma and lengthened the branch leading to *Ichthyornis* by 5 Ma. Lastly, to account for uncertainty in fossil tip dates, we randomly sampled the age of each fossil tip between the ages of its first and last occurrences 10 times, to produce 10 phylogenies with identical topologies (Supplementary Fig. S3a) but different tip dates for extinct taxa. To further investigate the effect that different hypotheses of broad-scale relationships within Neoaves had on results we also ran all analyses on the informal supertree constructed by Knapp et al. (2025), based on the Stiller et al. (2024) phylogeny (Supplementary Fig. S3b).

### Summarizing region shape variation using factor analysis

**Fig. 1:**
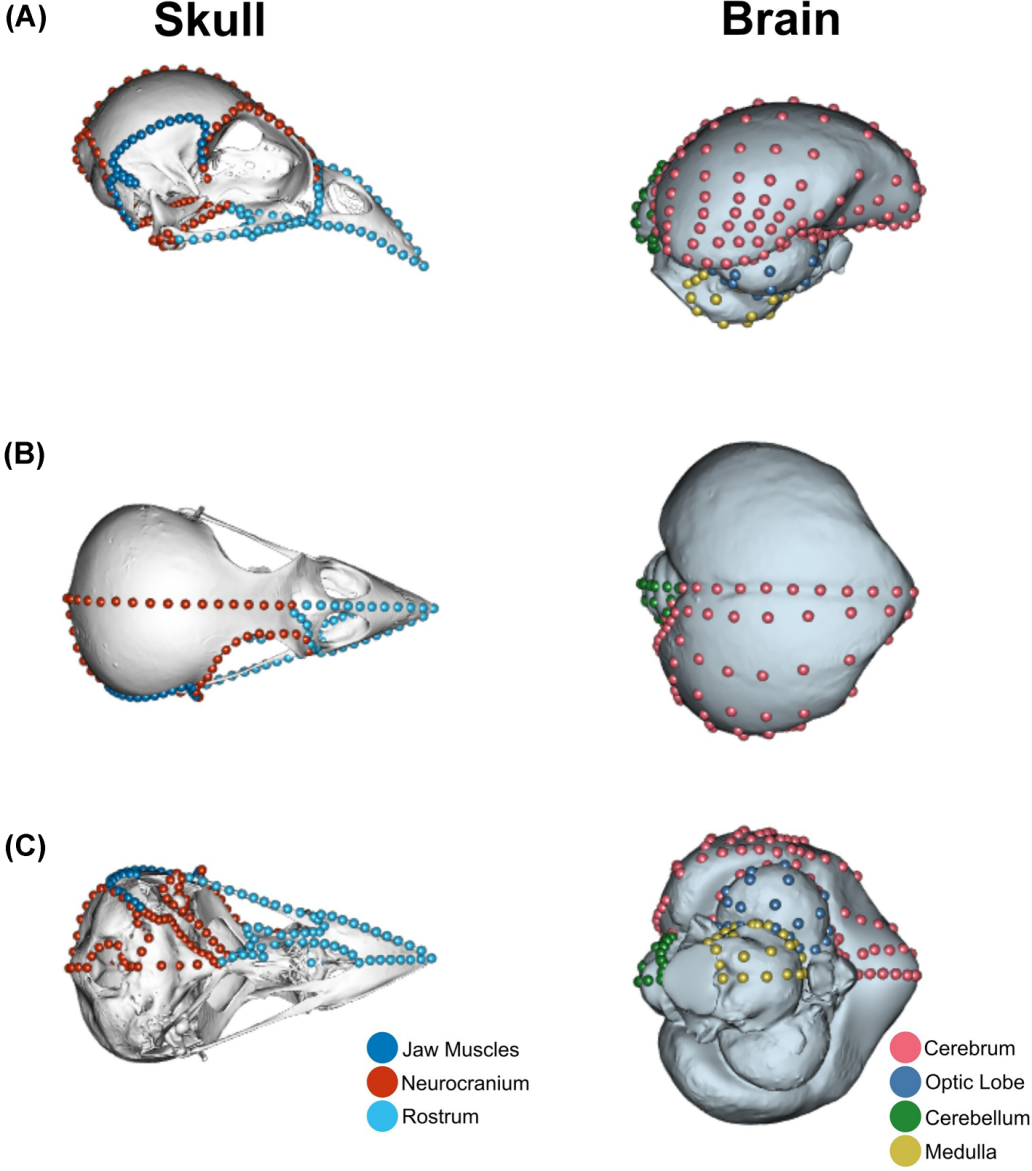
Landmarks used to characterize anatomical regions of the avian skull and brain. Landmarks are shown in A: lateral, B: dorsal, and C: ventral views. Reference landmarks and meshes are for the house sparrow (*Passer domesticus*).

We used FA, to define variables that captured covariation within each anatomical region (Fig. 1). FA is comparable to PCA in that both techniques are used to reduce the dimensionality of datasets for analysis and interpretation, with some key differences. PCA summarizes variance in the data into orthogonal components (David Polly et al. 2013; Greenacre et al. 2022). It is an exploratory technique which defines orthogonal axes which maximize variance, making it well suited as a method of ordination analysis for visualising high dimensional shape data. FA defines latent constructs which predict covariance between sets of variables. While exploratory factor analysis (EFA) can be used to maximize the covariance predicted by each successive latent factor (analogous in approach to PCA), confirmatory factor analysis (CFA) is an approach which tests the ability of underlying factors to explain covariation between sets of observed variables defined *a priori* (e.g., due to a hypothesized common underlying cause). It is important to note that while EFA’s will always fit the data better than theoretical models by design, the resulting factors are difficult to interpret biologically and make distinguishing between competing hypotheses difficult (Zelditch 1987).

We implemented all phylogenetic FAs and phylogenetic structural equation models using the R package *phylosem* (Thorson and van der Bijl 2023). We first used phylogenetic EFA, to explore shape variation within each landmark partition (rostrum, neurocranium, jaw muscles and endocast). EFA was chosen for the FA of these partitions in order to maximize the amount of covariance captured by the variables representing anatomical regions, as well as facilitate comparison with a more traditional PCA. In each case, covariance between landmarks within each partition was represented as loadings on between 1 and 4 latent factors. In order to ensure models were identifiable while not scaling the covariances to an arbitrary loading, we fixed the variance of each factor to be 1. This left the variance of each landmark and their covariances with each factor as free parameters to be estimated by the model. Additionally, in order to avoid nonidentifiability in the multiple factor EFAs, we dropped one landmark from each subsequent factor after the first (first landmark dropped from factor 2, landmarks 1 and 2 dropped from factor 3, landmarks 1, 2, and 3 dropped from factor 4). We also carried out separate CFA analyses on the endocast dataset in order to explore covariance patterns between the skull and regions of the brain linked to different functions, as well as between brain regions. For these analyses, we specified 1 factor corresponding to each major region of the brain (cerebrum, optic lobe, medulla, and cerebellum) as well as 1 factor each for the neurocranium, rostrum, and jaw muscles. Model functions were optimized with 5 loops of the quasi-Newton, gradient-based *nlminb* optimizer, followed by 5 loops of *phylosem’s* Newton optimizer to ensure minimisation of the gradient of the objective function. We specified a maximum of 10,000 evaluations of the objective function and 10,000 iterations. We checked convergence for each model by ensuring the Hessian matrix was positive definite (i.e., that the gradient of the marginal log-likelihood is a local minimum and increases in all directions), that all standard errors were positive and that the absolute value of the gradient of the marginal log-likelihood was < 0.01 for all parameters. Lastly, we performed PCA of the Procrustes-aligned coordinates for the skull and endocast as well as each landmark subset for each using the function “gm.prcomp” in the R package *geomorph* (Baken et al. 2021; Adams et al. 2025).

### Analyzing covariation between regions using structural equation models

We used phylogenetic structural equation models (phyloSEMs) to test different hypotheses of modularity and integration between the phylogenetic factors representing regions of the avian skull and brain (see Supplementary Methods). These corresponded to 14 hypotheses describing different organization of sets of traits into modules which influence each other (Table 1). The strength and directionality of relationships between variables in each SEM are represented by path coefficients, which are regression coefficients (Beta weights in the case of standardized variables). For example, a path coefficient of 0.5 means that 1 standard deviation unit of change in the predictor can explain a 0.5 standard deviation unit increase in the response variable. Absolute values of path coefficients can be greater than 1 when there are multivariate relationships, although this often reflects collinearity between variables. As the variables in our models represent biological constructs these collinear relationships can be informative, and we did not drop variables to reduce collinearity. Our models tested interactions between several of 6 hypothesized modules specified by latent variables; a “Brain” module influencing separate brain regions, a “Jaw” module influencing jaw muscle attachment sites (jaw MS) and rostrum shape (RS), a “Cranial” module influencing shape of the brain and neurocranium, a “Skull” module influencing jaw MS and neurocranium shape and a “Hard Tissue” module influencing neurocranium and RS. Lastly, we also specified a model where traits in the head were fully integrated without any modularity, as a “Single Module” influencing all traits. We also tested models without these modules represented by latent variables, corresponding to the 12 hypotheses outlined in Knapp et al. (2025). We used latent variables with a fixed variance of 1 to represent hypothesized modules which covaried with the factors representing traits which formed the module in question. We then represented the effect of allometry with a latent size variable which described how the measured variables of bodymass and brain volume covary. We ran four different versions of each model to investigate allometric patterns, with the latent size variable to represent allometry, with the direct effect of bodymass only, with the direct effect of endocranial volume only and without the influence of allometric scaling. This was to test whether models explicitly informed by allometric scaling better explain trait interactions and whether allometry affects different shape modules in different ways. We ran SEMs on the mean-centered raw variables as well as standardized variables (mean-centered with a fixed variance of 1). We ran each model on both sets of phylogenies described above, with 5 nlminb loops and 5 newton loops each with a maximum of 10,000 iterations. We then used the marginal Akaike information criterion to identify which models were most parsimonious. We note that marginal AIC is more appropriate than conditional AIC for cases (such as ours) where we seek to identify a parsimonious estimate for parameter linking traits (Zheng et al. 2024, Nov 21). We used AIC weights (the probability that a given model in the set is the best model in the set) to determine model performance with higher values indicating a better fit to the data. AIC weights < 0.05 therefore indicate the model is unsupported. We also used the difference in AIC between the best supported model and a given model in the set (ΔAIC) to evaluate whether other models performed similarly to the best model in each case. We follow the convention that ΔAIC < 2 indicates strong support, ΔAIC < 5 indicates moderate support and ΔAIC > 10 indicate the model is unsupported.

**Table 1:** Models of correlated evolution and modularity tested with *phyloSEM*.

Model name	Source	Summary of relationships
Jaw Module 1 (JM 1)	This paper	Brain is influenced by neurocranium and a jaw module of jaw MS and rostrum.
Two Modules 1 (TM 1)	This paper	Both cranial module of brain and neurocranium and jaw module of jaw MS & rostrum do not directly interact.
Two Modules 2 (TM 2)	This paper	Cranial module of brain & neurocranium influences jaw module of jaw MS & rostrum.
Two Modules 3 (TM 3)	This paper	Jaw module of jaw MS & rostrum influences cranial module of brain & neurocranium.
Brain Only 1 (BO 1)	This paper	All other regions influence brain independently. No allometric scaling effect on rostrum shape.
Brain Only 2 (BO 2)	This paper	All other regions influence brain independently. No allometric scaling effect on rostrum or jaw MS.
Single Module (SiM)	This paper	All regions influenced by a single module.
Skull Module 1 (SM 1)	This paper	Rostrum and skull module of jaw MS and neurocranium influence brain.
Skull Module 2 (SM 2)	This paper	Brain and rostum influence skull module of jaw MS and Neurocranium.
Jaw Module 2 (JM 2)	This paper	Brain and neurocranium influence jaw module of Jaw MS & rostrum.
Jaw Module 3 (JM 3)	This paper	Brain and jaw module of jaw MS & rostrum influence neurocranium.
Hard Tissue Module 1 (HTM 1)	This paper	Jaw MS and hard tissue module of rostrum & neurocranium influence brain.
Hard Tissue Module 2 (HTM 2)	This paper	Brain and jaw MS influence hard tissue module of neurocranium & rostrum.
Hard Tissue Module 3 (HTM 3)	This paper	Brain and hard tissue module of rostrum and neurocranium influence jaw MS.
Hand in Glove 1 (HG 1)	Knapp et al. (2025)	Brain influences neurocranium. Neurocranium influences rostrum & jaw MS.
Hand in Glove 2 (HG 1)	Knapp et al. (2025)	Brain influences neurocranium. Neurocranium influences rostrum and Jaw MS. Reciprocal interactions between all correlated traits.
Spatial Packing 1 (SP 1)	Knapp et al. (2025)	Brain influences neurocranium, rostrum and jaw MS.
Spatial Packing 2 (SP 2)	Knapp et al. (2025)	Reciprocal interactions between the brain and each other trait.
Spatial Packing 3 (SP 3)	Knapp et al. (2025)	Brain directly influences all traits except jaw MS.
Functional Matrix 1 (FM 1)	Knapp et al. (2025)	All other traits influence neurocranium.
Functional Matrix 2 (FM 2)	Knapp et al. (2025)	All other traits influence neurocranium. Reciprocal interactions between all traits other than neurocranium.
Ecological Selection 1 (ES 1)	Knapp et al. (2025)	Rostrum influences brain. Brain influences neurocranium. Neurocranium influences jaw MS.
Ecological Selection 2 (ES 2)	Knapp et al. (2025)	Rostrum influences neurocranium. Neurocranium directly influences Brain and jaw MS individually.
Ecological Selection 3 (ES 3)	Knapp et al. (2025)	Rostrum influences jaw MS and brain. Brain influences neurocranium.
Modular	Knapp et al. (2025)	Neurosensory elements and those associated with feeding are separate, integrated modules that do not interact with one another.
Fully integrated (FI)	Knapp et al. 2025	The most parameterized model, all traits interact.

*Note:* Abbreviations for model names are given in brackets after the model name in the names column.

## Results

**Fig. 2:**
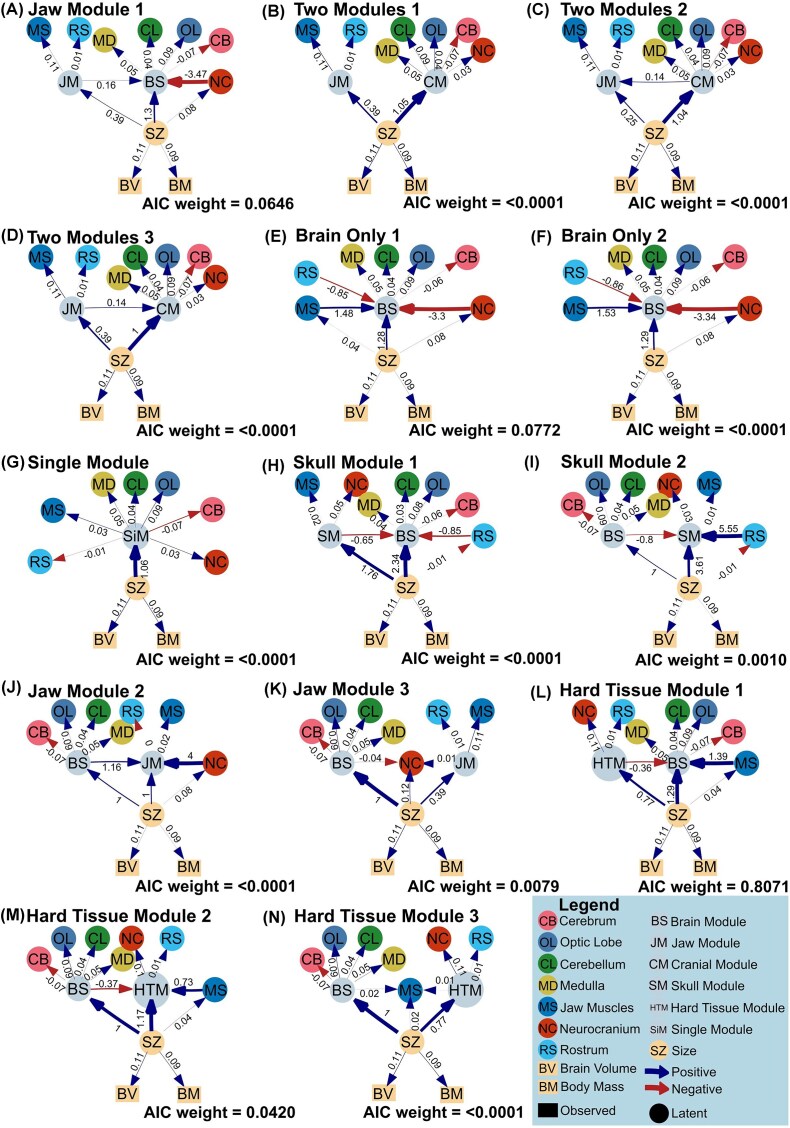
*phyloSEM* models of correlated trait evolution testing hypotheses of modularity and the effect of allometry on patterns of integration between the cranium and brain in birds. Arrows represent the correlations between variables. Numbers next to each arrow represent their path coefficients with the thickness of the arrows representing the strength of the covariances. AIC weights represent the conditional probability that a given model has the greatest support in the set of models tested.

AIC weights indicated *phyloSEM* Model Hard Tissue Module 1 with a latent variable for size was the best supported model (Fig. 2L: AIC 4316, AIC weight 0.8071), highlighting complex patterns of modularity among the skull, face, and braincase that were strongly influenced by allometric scaling. This model also included the four individual regions of the brain covarying as subunits of a brain module. This is supported by the results of FA, where EFA models that did not define the modular organization of the brain had consistently higher AIC values than those which defined factors based on brain regions (Supplementary Table S1). Two alternative models indicating a different pattern of relationships could be supported by our data, although showing substantially lower support than the Hard Tissue Module 1. Specifically, Brain Only 1, the model in which rostrum, neurocranium, and jaw muscle shape all independently affected brain shape but did not covary except with size (Fig. 2E: AIC 4317, AIC weight 0.0772) and Jaw Module 1, where the jaw module and neurocranium instead influenced variation in the brain module (Fig. 2A: AIC 4321, AIC weight 0.0646). Models which hypothesized direct relationships between anatomical regions received poor support (Supplementary Fig. S2).

These results remain largely robust regardless of the phylogeny (Table 2) and data treatment used (Supplementary Table S2), although support for the neurocranial and brain modules is stronger than for modules including the rostrum or jaw muscles. Similar results were recovered when the variances of each variable were unstandardized (Supplementary Table S2). Standardizing variance primarily affected the variance of the latent size variable representing allometry (due to the differing units of measurement for body mass and brain volume) and therefore the relative magnitude of the path coefficients between size and other modules.

**Table 2:** AIC Support for SEMs of modularity on different phylogenies.

	Claramunt et al. (2024)			Stiller et al. (2024)		
Rank	Model	ΔAIC	AIC weight	Model	ΔAIC	AIC weight
1	HTM 1	0	0.8071	HTM 1	0	0.7628
2	BO 1	4.6952	0.0772	BO 1	3.402	0.1392
3	JM 1	5.0512	0.0646	JM 1	4.285	0.0895
4	HTM 2	5.914	0.0420	HTM 2	9.142	0.0079
5	JM 3	9.2423	0.0079	JM 3	15.223	0.0004
6	SM 2	13.329	0.0010	ES2	16.718	0.0002
7	ES2	16.78	0.0002	HTM 3	21.569	<0.0001
8	ES1	23.966	<0.0001	JM 2	23.848	<0.0001
9	SM 1	24.643	<0.0001	FM1	24.754	<0.0001
10	SP1	24.954	<0.0001	ES1	25.654	<0.0001
11	FM1	28.51	<0.0001	ES3	25.67	<0.0001
12	HTM 3	29.323	<0.0001	Modular	25.843	<0.0001
13	ES3	29.67	<0.0001	SM 1	34.763	<0.0001
14	JM 2	33.959	<0.0001	JM 2	36.948	<0.0001
15	Modular	36.088	<0.0001	FI	42.013	<0.0001
16	BO 2	44.203	<0.0001	FM2	42.539	<0.0001
17	FM2	52.695	<0.0001	SM 2	154.94	<0.0001
18	FI	52.695	<0.0001	TM 3	154.94	<0.0001
19	TM 3	145.65	<0.0001	TM 1	157.57	<0.0001
20	TM 1	148.41	<0.0001	TM 2	158.69	<0.0001
21	SiM	149.1	<0.0001	SiM	163.27	<0.0001
22	TM 2	149.35	<0.0001	SP2	186.78	<0.0001
23	HG1	176.75	<0.0001	HG2	190.04	<0.0001
24	SP2	180.07	<0.0001	HG1	193.39	<0.0001
25	HG2	180.52	<0.0001	SP3	193.45	<0.0001
26	SP3	183.31	<0.0001	SP1	750.91	<0.0001

*Notes:* Differences in marginal AIC between each model and the best supported model (ΔAIC), as well as corresponding AIC weights. AIC weights can be interpreted as the conditional probability that the model is the best in the set of models. ΔAIC values > 5 and AIC weights < 0.05 therefore indicate little to no support relative to alternative models. Results are based on the Claramunt et al. (2024) and Stiller et al. (2024) bird phylogenies.

**Fig. 3:**
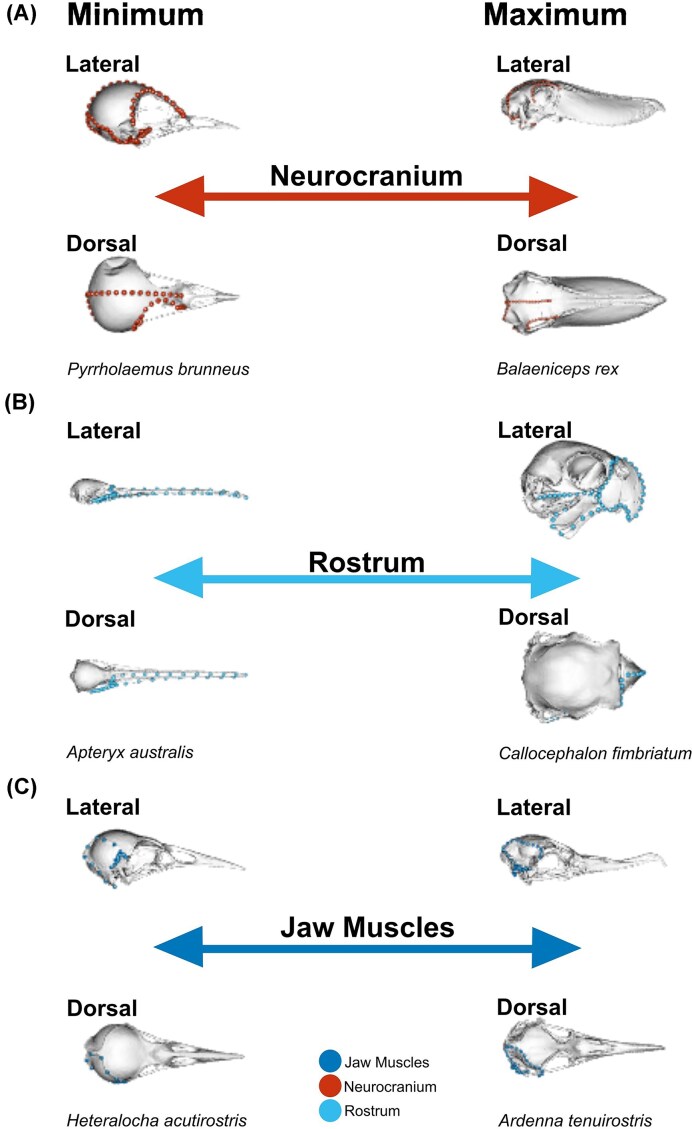
Variation described by latent variable factors for each anatomical region of the skull. (A) neurocranium shape (NC), (B) RS, (C) jaw muscle attachment sites (MS). In each case, taxa shown had the lowest (left) and highest (right) scores for the factor.

**Fig. 4:**
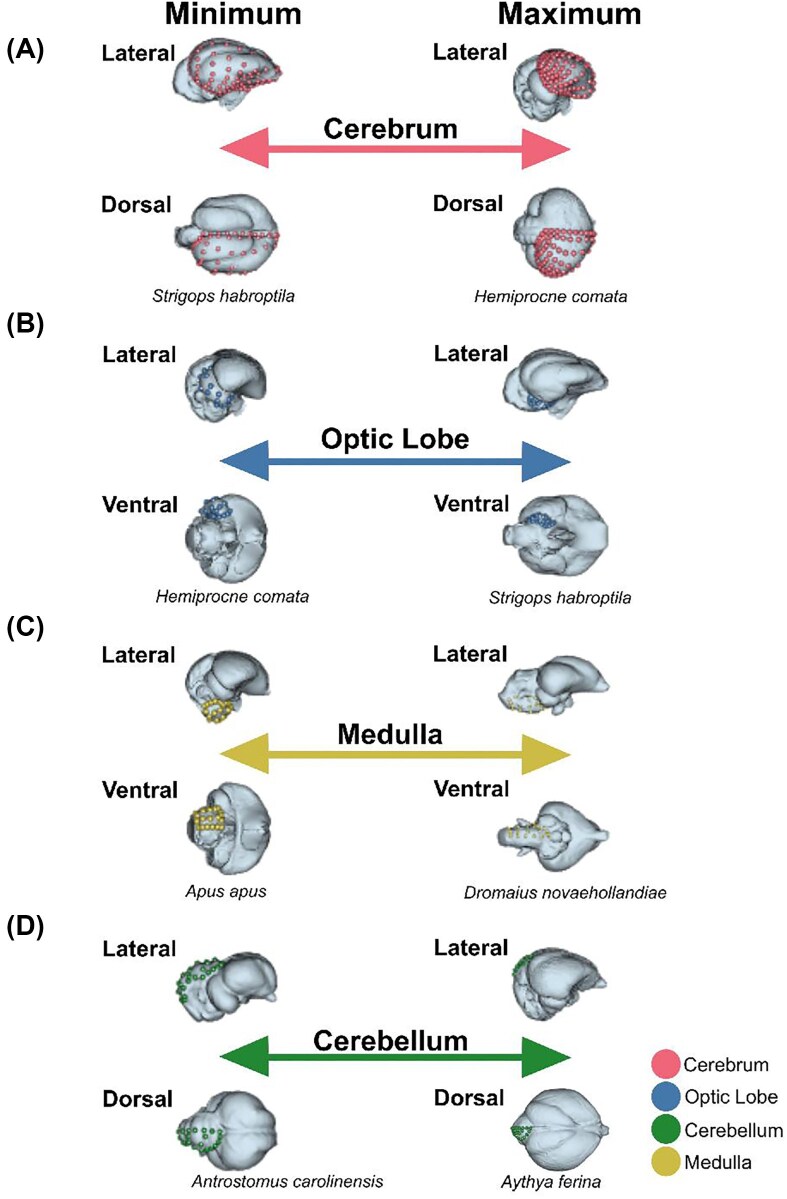
Variation described by latent variable factors for each anatomical region of the brain. (A) cerebrum shape (CB), (B) optic lobe shape (OL), (C) medulla shape (MD), (D) cerebellum shape (CL). In each case, taxa shown had the lowest (left) and highest (right) scores for the factor.

The influence of neurocranial shape on brain shape seems to correspond to the major components of shape variation in the avian skull and is largely associated with increases in flexion and orbit size (Fig. 3A). Cranial shape covaries negatively with brain shape, with increased orbit size and more brachycephalic neurocrania corresponded to a relatively smaller cerebrum (Fig. 4A) and larger, more globular optic lobes (Fig. 4B). These changes in cranial shape also tend to correlate with a smaller, more posteriorly compressed medulla (Fig. 4C). To a lesser degree, decreases in the basicranial angle with more brachycephalic braincases is also associated with a larger cerebellum (Fig. 4D) and corresponding shifts in the position of the brainstem. Skulls with more globular neurocrania and larger orbits also tend to have shorter, broader rostra (Fig. 3B), although overall skull shape only weakly covaries with RS (path coefficient: 0.11). Jaw muscle shape was also found to affect overall brain shape, with expansion of the cerebrum and optic lobes being associated with expansion of the adductor musculature (Fig. 3C), although this covariation is also relatively weak (path coefficient: 0.17).

PCA components were broadly in agreement with the variation described by PFA factors. For the neurocranium (Supplementary Fig. S4), PC1 (25.4% of variation) was associated primarily with increased cranial doming and large closely spaced orbits, while PC2 (16.5% of variation) described broadening of the braincase. For the rostrum (Supplementary Fig. S5), the main axis of variation was elongation (PC1 45.8%) followed by deepening and curving (PC2 14%). PCA analysis of jaw muscle shape (Supplementary Fig. S6) recovered deepening of the adductor attachment site as the greatest source of variation (PC 1 49.8%), followed by broadening of the adductor (PC 2 13.2%). Lastly, the major components of variation in brain shape (Supplementary Fig. S7) were found to be the relative size of the cerebrum and change in basicranial angle (PC 1 33.4%), as well as elongation of the whole brain (PC 2 25.3%).

Allometry has a marked effect on the shape variation of different regions, regardless of the interaction between modules. Models including covariation between size and shape were consistently better supported than equivalents that did not include these relationships (Table 3). Critically, when allometric effects are excluded we recover support for different patterns of modularity with integration between the neurocranium and jaw musculature in a “skull” module separate from rostral shape variation (Table 2, no size column). Support for relationships between these modules was ambiguous, with a model in which the brain module is influenced by the skull module and rostrum (Model SM 1: AIC 4646, AIC weight 0.535) and a model in which the skull module is influenced by the brain module and rostrum (Model SM 2: AIC 4646, AIC weight 0.463) receiving roughly equal support. Models in which a single proxy of size (either bodymass or brain volume) directly influenced shape variables instead of specifying a latent variable for allometry produced similar results in most cases (Table 2), albeit with weaker support (bodymass—Models BO 1: AIC weight 0.4707; JM 1: AIC weight 0.2698; HTM 1: AIC weight 0.2553. Brain volume–Models HTM 1: AIC weight 0.5610; BO 1: AIC weight 0.3351; HTM 2: AIC weight 0.0706). Brain volume was found to have a weaker effect on shape variation than body size for all skull components, but a slightly stronger effect on brain shape (Supplementary Fig. S1). Brain shape was found to show the strongest allometric variation however (path coefficients of 1.0–1.3), resulting in a slightly greater proportion of the variance in brain volume being attributed to size effects (path coefficient 0.11 for brain volume vs. 0.09 for body size). In contrast, both jaw muscle and RS showed minimal covariance with allometry (jaw muscle: 0.04, rostrum: −0.01).

**Table 3:** AIC Support for SEMs modeling covariance with bodymass, brain volume, and not modeling allometric covariance (no size).

	Bodymass only			Brainvolume only			No size		
Rank	Model	ΔAIC	AIC weight	Model	ΔAIC	AIC weight	Model	ΔAIC	AIC weight
1	BO 1	0	0.471	HTM 1	0	0.5610	SM 1	0	0.5353
2	JM 1	1.1130	0.2710	BO 1	1.0306	0.3351	SM 2	0.2845	0.4643
3	HTM 1	1.2237	0.2553	HTM 2	4.1464	0.0706	BO 1	17.1887	0.0001
4	HTM 2	10.1192	0.0030	SM 1	7.3842	0.0140	BO 2	17.1887	0.0001
5	JM 3	13.4475	0.0006	JM 3	7.47485	0.0134	SP2	17.1887	0.0001
6	SM 2	14.3619	0.0004	SM 2	9.3550	0.0052	TM 3	19.7181	<0.0001
7	ES2	14.9902	0.0003	ES2	13.0926	0.0008	TM 2	19.7190	<0.0001
8	HTM 3	17.6632	0.0001	ES1	20.6660	<0.0001	TM 1	19.7191	<0.0001
9	ES1	20.2537	<0.0001	FM1	24.7769	<0.0001	HTM 3	19.8059	<0.0001
10	JM 2	20.4381	<0.0001	ES3	25.9804	<0.0001	HTM 1	25.6113	<0.0001
11	FM1	25.3463	<0.0001	HTM 3	29.3187	<0.0001	JM 2	26.8056	<0.0001
12	ES3	26.5577	<0.0001	JM 2	31.9547	<0.0001	ES2	27.1223	<0.0001
13	BO 2	32.2098	<0.0001	Modular	32.6416	<0.0001	ES1	27.2412	<0.0001
14	Modular	32.9933	<0.0001	BO 2	40.7650	<0.0001	JM 1	30.9400	<0.0001
15	FM2	39.7324	<0.0001	FM2	50.6911	<0.0001	HG1	32.5378	<0.0001
16	FI	39.7324	<0.0001	FI	50.6911	<0.0001	ES3	35.6174	<0.0001
17	SP2	130.765	<0.0001	JM 1	113.004	<0.0001	JM 3	37.3808	<0.0001
18	HG1	134.998	<0.0001	TM 3	145.637	<0.0001	FM1	37.5695	<0.0001
19	HG2	137.445	<0.0001	TM 1	145.637	<0.0001	HG2	39.5690	<0.0001
20	SP3	141.113	<0.0001	TM 2	145.637	<0.0001	HTM 2	40.9933	<0.0001
21	SiM	157.224	<0.0001	HG1	182.794	<0.0001	FM2	46.1938	<0.0001
22	TM 1	157.303	<0.0001	HG2	186.360	<0.0001	FI	46.1938	<0.0001
23	TM 3	157.303	<0.0001	SP3	191.329	<0.0001	SP3	47.6567	<0.0001
24	SM 1	256.052	<0.0001	SP2	192.104	<0.0001	Modular	51.0549	<0.0001
25	SP1	688.570	<0.0001	SiM	733.602	<0.0001	SiM	496.432	<0.0001
26	TM 2	776.634	<0.0001	SP1	733.602	<0.0001	SP1	496.432	<0.0001

*Notes:* Differences in marginal AIC between each model and the best supported model (ΔAIC), as well as corresponding AIC weights. AIC weights can be interpreted as the conditional probability that the model is the best in the set of models. ΔAIC values > 5 and AIC weights < 0.05 therefore indicate little to no support relative to alternative models. Results are based on the Claramunt et al. (2024) bird topology.

## Discussion

We found strong evidence that shape variation of the avian head follows a modular pattern, in agreement with previous studies of birds and non-avian theropods (Balanoff, Smaers and Turner 2016; Felice et al. 2019; Watanabe et al. 2021) The shape of the skull and face influence the shape of major brain regions (cerebrum, medulla, optic lobe and cerebellum) differentially and there is stronger integration between anatomical regions (i.e., brain, neurocranium, rostrum and jaw muscles) and weaker integration between functional regions (i.e., the cranial module of brain and neurocranium, the jaw module of jaw muscles and rostrum). Our analyses found greatest support for models of trait evolution where brain shape is influenced by the shape of both the neurocranium and jaw musculature (Model HTM 1, Fig. 2, and Table 2). In contrast, models representing the opposite pattern, with brain shape evolution influencing neurocranium and jaw shape evolution, were much less well supported (e.g., Models HTM 2, JM 3, SM 2, HTM 3 and JM 2 as well as HG1, HG2 and SP1, Table 2). This is surprising given that signaling in the formation of the brain influences growth of the skull roof and face (Moss and Young 1960; Richtsmeier and Flaherty 2013; Fabbri et al. 2017). In all vertebrates, the brain plays a key role in the morphogenesis of the head during early embryological development (Marcucio et al. 2011), which is governed by highly conserved shared signaling pathways (Kim et al. 1998; Pan et al. 2013; Marchini et al. 2021). Adult skull morphology is therefore ultimately determined during embryogenesis and subsequent development (Conith et al. 2023), and selection on adult form acts upon differences in the timing and location of developmental signals (Bhullar et al. 2016; Fabre et al. 2020). Indeed, interspecific differences in cranial morphology can be seen soon after the formation of the embryonic chondrocranium (Hüppi et al. 2021).

We propose two potential mechanisms to explain why brain shape evolution is influenced by skull and muscle evolution in our models despite the well-known morphogenic primacy of the brain during embryonic growth. First, physical constraints can act to influence brain shape during later development. This is the basis of the spatial packing hypothesis, in which growth of the various structures in the head are influenced by the need to accommodate each other within a limited space (Jeffery and Mendias 2014; Jeffery et al. 2021). This effect has been observed during development, for example the correlation between increased cerebrum size and basicranial flexion observed in birds (Marugán-Lobón et al. 2022) and primates (Bastir et al. 2010). Our results support the spatial packing hypothesis as an evolutionary mechanism, implying that selection on neurocranium shape and jaw musculature influence brain shape later in development. Additional structural constraints imposed by a more rigid skull are likely also important in determining adult morphology. These seem to be the dominant interactions at the macroevolutionary level, suggesting subsequent interactions may overwrite the signal which results from neural patterning in early embryogenesis.

Second, whereas growth of the skull roof and face depend on the molecular signals arising from the developing brain, other skull regions are less dependent on the brain (Lieberman et al. 2000). For example, basicranial growth may be regulated intrinsically to a greater degree than other parts of the face and skull (Scott 1958) and reaches adult size earlier in ontogeny (Bastir et al. 2006). Growth of the basicranium may therefore influence overall skull and brain growth, most notably by influencing the basicranial angle (Lieberman et al. 2008). As the neck and head are functionally integrated in birds (Marek and Felice 2023; Marugán-Lobón and Nebreda 2025), there are likely strong selective pressures to accommodate shape changes in the base of the cranium close to the occipital condyle. Likewise, feeding imposes another functional constraint on the head. The jaws arise early in development from the mandibular and maxillary primordia, showing rapid changes in growth trajectories which can account for later morphological divergences (Young et al. 2014). Studies have shown regulation is also highly intrinsic and established locally within the jaw skeleton, as ducks with quail donor neural crest mesenchyme generate short, blunt, quail-like beaks (Schneider 2026). Previous work has also found that chicken jaw adductor muscle size and brain size show a significant (although weak) negative correlation when head width is accounted for (Cerio et al. 2023).

In addition, we emphasize the patterns reconstructed here represent evolutionary (among species) correlations, not static (within population) or developmental (within growth series) correlations. Although we have focused on how developmental factors might shape the evolutionary correlations among the brain, skull, and jaw muscles, this is only one of many ways that evolutionary integration can arise. For example, under neutral evolution (genetic drift, fluctuating selection) traits that share genetic correlations are expected to show evolutionary correlations as well (Revell and Harmon 2008). Alternatively, evolutionary correlations can arise in traits that are co-selected, even if they do not share developmental or genetic correlations (Badyaev 2010; Sol et al. 2016). For example, species adapted for cold climates might exhibit correlated evolution of fur color and body mass even if there is no genetic correlation between these traits (Felsenstein 1988). Although we did not model natural selection here (for example, using Ornstein–Uhlenbeck processes), co-selection could generate macroevolutionary patterns of trait variation and covariation that are overprinting fine-scale developmental patterns.

Critically, patterns of interactions between modules are affected by allometry, as we found that an effect of cranium shape on brain shape was dependent on modeling the effect of allometric covariation between traits. Our models which included a latent “size” variable characterizing allometric scaling variation were better supported than models which did not consider allometric effects, as well as those models which only considered the direct effects of either brain volume or body mass independently. Different aspects of allometry seem to correlate with some regions of the head more strongly than others. While brain volume is more strongly correlated with brain shape, body mass exhibits a stronger correlation with skull and jaw muscle shape. Body size is known to correlate with several ecological and life history traits in birds (Olson et al. 2009), as well as being a strong predictor of beak morphology (Bright et al. 2016; Friedman et al. 2019). Models which did not consider allometric effects more strongly supported brain shape influencing cranium shape (Model SM 2), consistent with expected patterns resulting from the morphogenic primacy of the brain relative to the cranium.

The differences in support between models which include an effect of allometric scaling on relationships between modules and those that do not suggests that some of the potential effect of brain shape on the cranium can be explained by allometric covariance. Brain shape is known to show significant allometric variation in birds (Kawabe et al. 2013), largely associated with increased flattening and elongation at larger sizes. These patterns could be the result of selective pressures to maintain relatively large brains at small sizes necessitating increased flexion of the brain and basicranium. Both the cerebrum and optic lobes appear to show stronger covariance with overall brain shape and thus greater integration with the head as a whole. In the case of the optic lobes, this likely reflects the importance of both the optic lobes and orbits in the visual sensory system. Patterns of shape variation in the cerebrum contrasted with those of the rest of the brain but correlated with the neurocranium, perhaps indicating structural or developmental trade-offs between regions of the brain and integration between the pallium and cranial vault. While the cerebrum tended to increase in size through lateral expansion, midbrain regions tended to elongate, resulting in flexion of the brain and associated basicranium. Both of these regions are also among the last to mature in the brain (Charvet and Striedter 2009), increasing the potential for interactions with the developing cranium.

While support was consistent for interactions between a brain module and some degree of integration between skull regions, support for the direction of interactions (or indeed whether interactions are reciprocal) was more ambiguous. This may reflect trade-offs between the skull and brain as well as the potential influence of other soft tissues in the head, particularly later in ontology. The eyes, similar to the brain, arise early in development and there is evidence that orbit and brain shape closely covary in birds (Kawabe et al. 2013). Conversely, we found that patterns of shape variation in the rostrum and, to a lesser degree, jaw musculature appeared somewhat decoupled from those in the rest of the skull and brain. Species of Caribbean bullfinches show morphologically similar bills generated using different developmental programs (Mallarino et al. 2012), suggesting that the rostrum may be less subject to developmental constraints. The influence of different soft tissues also changes throughout development. This creates the potential for heterochronic shifts which could influence long term evolutionary patterns and account for the influence of allometry on covariance between the brain and skull. Specifically, competition between the jaw muscles and other aspects of the head may be limited to the perinatal period when they are relatively large, while negative allometry between the brain and eyes is explained by growth of the brain postnatally (Cerio et al. 2023). The relative growth of the cranial bones and timing of suture fusion has also been found to have an impact on later brain development (Richtsmeier et al. 2006; Richtsmeier and Flaherty 2013). The patterns of modularity found in this study could therefore represent “overprinting” of earlier known patterns of cranial formation established by development of the brain. These subsequent interactions could be either biomechanical at the interface between both structures (as in the functional matrix hypothesis) or interaction with other soft tissue structures (as in the spatial packing hypothesis).

We highlight *phyloSEM* as a useful tool for studying modularity and integration as it allows a system of complex hierarchical relationships to be directly investigated in the same model. The various structures within the avian head all show some degree of integration both developmentally and structurally. We have shown how SEM can account for these complex relationships by allowing reciprocal and indirect relationships between variables to be modeled. SEM also allows for separate sets of unlinked relationships to be evaluated simultaneously, as in cases where structures within modules are expected to covary but the modules themselves are entirely independent. Most importantly, SEM allows for the inclusion of unmeasured latent variables which allows for hierarchical models with multiple levels of modularity, such as where brain regions covary as parts of an integrated structure (the brain), which in turn covaries with other modules.

*phyloSEM* models of covariation of traits in the head across the phylogeny of birds supports a model where the avian brain shape is most strongly influenced by the shape of the neurocranium, but where attachment sites of the jaw musculature also have an effect on brain shape. This suggests that, on the macroevolutionary scale, the brain and skull of birds evolve as a hierarchy of co-evolving traits, where the dominant signal results from complex interactions between traits at later developmental stages. Patterns of integration differ greatly, particularly across the skull. The rostrum shows relatively weak integration with other regions, suggesting that functional considerations related to factors such as diet are also important. The inclusion of latent variables that characterize body and brain size variation in our SEM models makes it clear that allometry impacts regions of the skull and brain differently. This suggests that different anatomical modules are subject to different size constraints, impacting the interactions between them. Given the nature of the patterns recovered, it is likely that the degree and nature of integration varies not only over the course of development, but across the bird tree of life. More work is needed to investigate the impact of allometry on the interactions between modules and how these patterns may vary between major clades, such as the Oscines.

## Supplementary Material

icag106_Supplemental_File

## Data Availability

The data and software used in this study are available on GitHub: https://github.com/JWOyston/Bird-Brain-SEM.git

## Notes

From the symposium “Integrating integration: How cellular mechanisms of trait covariation shape evolutionary patterns of phenotypic integration” presented at the annual meeting of the Society for Integrative and Comparative Biology, January 6th, 2026.
