# Supplementary material for: Structural Equation Modeling Reveals How Allometry Shapes Integration in Avian Cranial Evolution
Source: Integr Comp Biol. 2026 Jul 2;66:icag106. doi: 10.1093/icb/icag106 (PMC13393896; doi:10.1093/icb/icag106)
Supplement: icag106_Supplemental_File [file icag106_supplemental_file.docx]

**Supplementary Methods**

**Summary of SEM Hypotheses**

**Models of modularity (this study)**

JM 1: Skull shape affects brain shape with brain regions forming one anatomical module and a hypothesised functional feeding module of Jaw Muscles & Rostrum.

TM 1: Two modules which do not directly interact. One consisting of the brain and braincase (neurocranium), the other consisting of the jaw muscles and rostrum.

TM 2: Two modules where the cranial module (brain & neurocranium) influences the jaw module (jaw muscles and rostrum).

TM 3: Two modules where the jaw module (jaw muscles and rostrum) influences the cranial module (brain & cranium).

BO 1: All skull regions (neurocranium, rostrum and jaw muscles) influence brain shape with brain regions forming one anatomical module. No allometric effect on rostrum shape.

BO 2: All skull regions (neurocranium, rostrum and jaw muscles) influence brain shape with brain regions forming one anatomical module. No allometric effect on rostrum shape or jaw muscle shape.

SiM: No modularity within the head but covariance between all regions. All skull and brain regions influenced by a single latent variable which in turn covaries with size.

SM 1: Rostrum and skull shape affects brain shape with brain regions forming one anatomical module and a hypothesised skull module consisting of Jaw Muscles and Neurocranium.

SM 2: Brain and rostral shape affect skull shape with brain regions forming one anatomical module and a hypothesised skull module consisting of Jaw Muscles and Neurocranium.

JM 2: Brain and neurocranium shape affect a hypothesised functional jaw module of Jaw Muscles & Rostrum.

JM 3: Brain and a hypothesised functional jaw module of Jaw Muscles & Rostrum affect neurocranial shape.

HTM 1: Jaw Muscle shape and a hypothesised hard tissue module consisting of Rostrum and Neurocranium affect brain shape.

HTM 2: Brain and Jaw Muscle shape affect a hypothesised hard tissue module consisting of Jaw Muscles and Neurocranium.

HTM 3: Brain and a hypothesised hard tissue module consisting of Jaw Muscles and Neurocranium affect jaw muscle shape.

**Models of direct interactions between traits (Knapp *et al.* 2025)**

HG1: Hand in glove 1. Brain shape influences neurocranium shape and neurocranium influences rostrum and jaw muscle shape.

HG2: Hand in glove 2. Brain shape influences neurocranium shape and neurocranium influences rostrum and jaw muscle shape. Two-way interactions between all correlated regions.

SP1: Spatial Packing 1. Brain shape directly influences neurocranium, rostrum and jaw muscle shape, with no direct interactions between other regions.

SP2: Spatial Packing 2. Brain shape directly influences each region, with reciprocal interactions between these regions and the brain.

SP3: Spatial Packing 3. Brain shape directly influences all other regions except jaw muscle shape, which is influenced by rostrum shape.

FM1: Functional Matrix 1. Neurocranial shape is influenced by all other regions which do not influence each other.

FM2: Functional Matrix 2. Neurocranial shape is influenced by all other regions. All other regions have two way interactions with each other but not with the neurocranium.

ES1: Ecological selection 1. Rostrum shape influences brain shape. Brain shape influences neurocranial shape. Jaw muscle shape is influenced by neurocranial shape.

ES2: Ecological selection 2. Rostrum shape influences neurocranium shape, which directly influences all other regions.

ES3: Ecological selection 3. Rostrum shape influences jaw muscle and brain shape. Brain shape influences neurocranium shape.

Modular: Two anatomical modules, Brain and neurocranium, Rostrum and jaw muscles with two way interactions between regions within modules but no direct interactions between modules.

FI: Fully integrated. All regions show two-way interactions with all other regions.

**Comparison of PFA and PCA Results**

Principal components analysis of the whole skull showed that elongation (PC 1) accounted for 45% of total shape variation. This axis distinguishes broad, flat short forms with short rostra at low PC1 values from narrow skulls with extremely long rostra at high PC1 values. Variation in rostrum shape accounts for the next greatest source of variation (PC 2: 11%). Taxa with low PC2 scores show shallow long rostra which are more curved, while high PC2 scores are associated with deeper, broader rostra which are both shorter and straighter. Lastly, PC3 accounts for around 9.5% of overall shape variation and describes differences in cranial shape. Low values of PC3 are associated with elongate, high and rounded (dolichocephalic) crania, while high values of PC3 are associated with broad, anteriorly flattened (brachycephalic) crania. For the neurocranium only, the first factor of the PFA is associated with relatively large orbits, increased doming and posterior expansion of the braincase and narrowing of the prefrontal bone. PFA factor 2 distinguishes angular crania with a more steeply sloping frontal from crania with a rounded parietal and squamosal region and curved frontals. Lastly, PFA factor 3 is associated with a broadening of the braincase and forward facing orbits. PCA components are in agreement with the variation described by PFA factors. On PC1 (25.4% of variation) high values are associated with increased cranial doming and large closely spaced orbits. The next greatest axis of variation (PC2: 16.5%) describes broadening of the braincase and steeper sloping of the frontal and prefrontal, followed by narrowing and flattening of the whole cranium (PC3: 8.5%). The greatest factor of covariation (PFA 1) in the rostrum is elongation. PFA 2 is associated with a curving and slight deepening of the rostrum, while PFA 3 describes broadening and straightening. PCA analysis shows strong agreement with these factors, with elongation on PC1 (45.8%), deepening and curving on PC2 (14%) and narrowing and strong curving on PC3 (10.4%). The first PFA factor for jaw muscle shape describes variation in the shape and area of the attachment sites for the adductor and depressor mandibulae, with a greatly expanded, deep attachment sites at low values (particularly for the adductor mandibulae) and broad, shallow attachment sites at high values. PFA 2 primarily reflects differences in the relative size of the attachment sites, with a greatly expanded adductor mandibulae attachment site at low values and equally sized, rounded attachment sites at high values. Lastly, PFA 3 describes differences in the shape of the attachment sites a more anterio-ventrally expanded, straight-margined adductor site and more rounded depressor site at high values. Principal components analysis recovers expansion and deepening of the adductor mandibulae as the greatest source of variation (PC 1: 49.8%), followed by reduction of the depressor and anterio-posterior expansion of the adductor (PC 2: 13.2%) and lastly expansion and rounding of the depressor with a relatively reduced, deepened adductor (PC 3: 7.4%).

For the endocast, factor 1 describes a relative reduction in cerebrum size as well as a reduction in curvature of the anterior of the brain and an overall lengthening and flattening of the brain. Factor 2 primarily describes a reduction in basicranial angle as well as flattening of the cerebrum and elongation of the cerebellum and medulla. Factor 3 describes a relative narrowing of the cerebellum and surrounding areas and a relative broadening of the cerebrum and optic lobes. The first 3 principal components for the endocast show broad agreement with PFA factors, with some differences in how the variance is partitioned. The first two principal components of the endocast describe similar proportions of variation in the dataset (PC1: 33.4%, PC2: 25.3%). PC1 describes relative reduction of the cerebrum and reduction and broadening of the mid and hindbrain associated with a decrease in the basicranial angle. PC2 describes brain elongation, with a narrowing of the cerebrum and lengthening of other brain regions at high PC2 values. PC3 (7.3% of total shape variation) primarily describes a change in angle and shape of the cerebrum, with higher values of PC3 associated with a less rounded, more anteriorly tapered cerebrum. Within most major brain regions (cerebrum, optic lobe and cerebellum), covariation is largely associated with a decrease in the relative size of the region in question. The exception to this is the medulla, where high values are associated with an increase in relative size. In the case of the cerebrum and optic lobes, this decrease is facilitated primarily through an decrease in width. In the cerebellum and medulla, changes in relative size decrease are accommodated through elongation, changing the orientation of the hindbrain and brainstem to be positioned more posteriorly, rather than ventrally under the brain. Descriptions of covariation from PFA and variation from PCA show broad agreement for the endocast as well as all component parts of the skull. This agreement is greatest between axes/factors which capture the greatest amount of shape disparity. Unless otherwise stated we therefore present results for models using a single latent variable (factor) to capture covariation within component parts of the skull and regions of the brain.

Supplementary Table 1: Model Support For Brain Region Modules

| Model | EFA (4 Factors) AIC | Region Modules AIC | ΔAIC |
| --- | --- | --- | --- |
| JM1 | 5156.91 | 4974.139 | 182.771 |
| TM 1 | 5176.894 | 4462.437 | 714.457 |
| TM 2 | 5178.215 | 4462.05 | 716.165 |
| TM 3 | 5175.594 | 4462.368 | 713.226 |
| BO 1 | 5166.786 | 4317.398 | 849.388 |
| BO 2 | 5192.376 | 4356.905 | 835.471 |
| SiM | 5178.185 | 4463.803 | 714.382 |
| SM 1 | 5293.818 | 4350.09 | 943.728 |
| SM 2 | 5292.02 | 4326.031 | 965.989 |
| JM 2 | 5262.813 | 4348.661 | 914.152 |
| JM 3 | 5160.031 | 4323.95 | 836.081 |
| HTM 1 | 5164.367 | 4316.425 | 847.942 |
| HTM 2 | 5292.02 | 4320.618 | 971.402 |
| HTM 3 | 5173.185 | 4345.965 | 827.22 |
| HG1 | 5322 | 4489.457 | 832.543 |
| HG2 | 5323.312 | 4493.22 | 830.092 |
| SP1 | 5327.823 | 5031.497 | 296.326 |
| SP2 | 10888.048 | 4492.775 | 6395.273 |
| SP3 | 5305.866 | 4496.01 | 809.856 |
| FM1 | 5331.615 | 4341.212 | 990.403 |
| FM2 | 5319.047 | 4367.386 | 951.661 |
| ES1 | 5174.075 | 4336.668 | 837.407 |
| ES2 | 5194.145 | 4329.483 | 864.662 |
| ES3 | 5164.183 | 4342.372 | 821.811 |
| Modular | 5210.531 | 4348.79 | 861.741 |
| FI | 5176.456 | 4367.399 | 809.057 |

Supplementary Table 2: Unstandardized & Standardized SEM Models

|  | Stiller et al. 2024 | |  |  | Claramunt et al. 2024 | |  |  |
| --- | --- | --- | --- | --- | --- | --- | --- | --- |
|  | Raw |  | Standardised | | Raw |  | Standardised | |
| Rank | Model | AIC | Model | AIC | Model | AIC | Model | AIC |
| 1 | HTM 1 | 12510.07 | HTM 1 | 4316.425 | HTM 1 | 14307.53 | HTM 1 | 4721.729 |
| 2 | BO 1 | 12511.04 | BO 1 | 4317.398 | BO 1 | 14310.73 | BO 1 | 4725.131 |
| 3 | JM 1 | 12513.4 | JM 1 | 4320.618 | JM 1 | 14310.96 | JM 1 | 4726.014 |
| 4 | HTM 2 | 12514.55 | HTM 2 | 4323.95 | HTM 2 | 14316.67 | HTM 2 | 4730.871 |
| 5 | JM 3 | 12517.59 | JM 3 | 4326.031 | JM 3 | 14322.09 | JM 3 | 4736.952 |
| 6 | SM 2 | 12519.67 | ES2 | 4329.483 | ES2 | 14322.28 | SM 2 | 4738.447 |
| 7 | ES2 | 12523.09 | HTM 3 | 4336.668 | HTM 3 | 14324.26 | ES2 | 4743.298 |
| 8 | ES1 | 12530.27 | JM 2 | 4341.212 | JM 2 | 14325.89 | ES1 | 4745.577 |
| 9 | FM1 | 12534.78 | FM1 | 4342.372 | FM1 | 14331.66 | SM 1 | 4746.483 |
| 10 | ES3 | 12536.26 | ES1 | 4345.965 | ES1 | 14332.4 | SP1 | 4747.383 |
| 11 | HTM 3 | 12539.66 | ES3 | 4348.661 | ES3 | 14334.9 | FM1 | 4747.399 |
| 12 | JM 2 | 12542.3 | Modular | 4348.79 | Modular | 14342.65 | HTM 3 | 4747.572 |
| 13 | Modular | 12542.35 | SM 1 | 4350.09 | SM 1 | 14343.65 | ES3 | 4756.492 |
| 14 | SM 1 | 12543.86 | BO 2 | 4356.905 | BO 2 | 14344.49 | JM 2 | 4758.677 |
| 15 | BO 2 | 12550.55 | FI | 4367.386 | FI | 14349.85 | Modular | 4763.742 |
| 16 | FI | 12560.96 | FM2 | 4367.399 | FM2 | 14349.87 | BO 2 | 4764.268 |
| 17 | FM2 | 12560.96 | SM 2 | 4462.05 | SM 2 | 14447.42 | FM2 | 4876.667 |
| 18 | TM 3 | 12655.52 | TM 3 | 4462.368 | TM 3 | 14462.51 | FI | 4876.669 |
| 19 | TM 1 | 12655.52 | TM 1 | 4462.437 | TM 1 | 14462.57 | TM 3 | 4879.295 |
| 20 | TM 2 | 12655.55 | TM 2 | 4463.803 | TM 2 | 14462.69 | TM 1 | 4880.419 |
| 21 | SiM | 12657.48 | SiM | 4489.457 | SiM | 14466.41 | SiM | 4884.998 |
| 22 | HG1 | 12683.1 | SP2 | 4492.775 | SP2 | 14494.34 | TM 2 | 4908.512 |
| 23 | SP2 | 12686.4 | HG2 | 4493.22 | HG2 | 14500.93 | HG1 | 4911.768 |
| 24 | HG2 | 12686.86 | HG1 | 4496.01 | HG1 | 14500.99 | SP2 | 4915.123 |
| 25 | SP3 | 12689.6 | SP3 | 4974.139 | SP3 | 14513.63 | HG2 | 4915.179 |
| 26 | SP1 | 13242.36 | SP1 | 5031.497 | SP1 | 15063.47 | SP3 | 4927.599 |

**Supplementary Figure 1: Effect of Size Variables on Correlations Between Pairs of Variables**

**
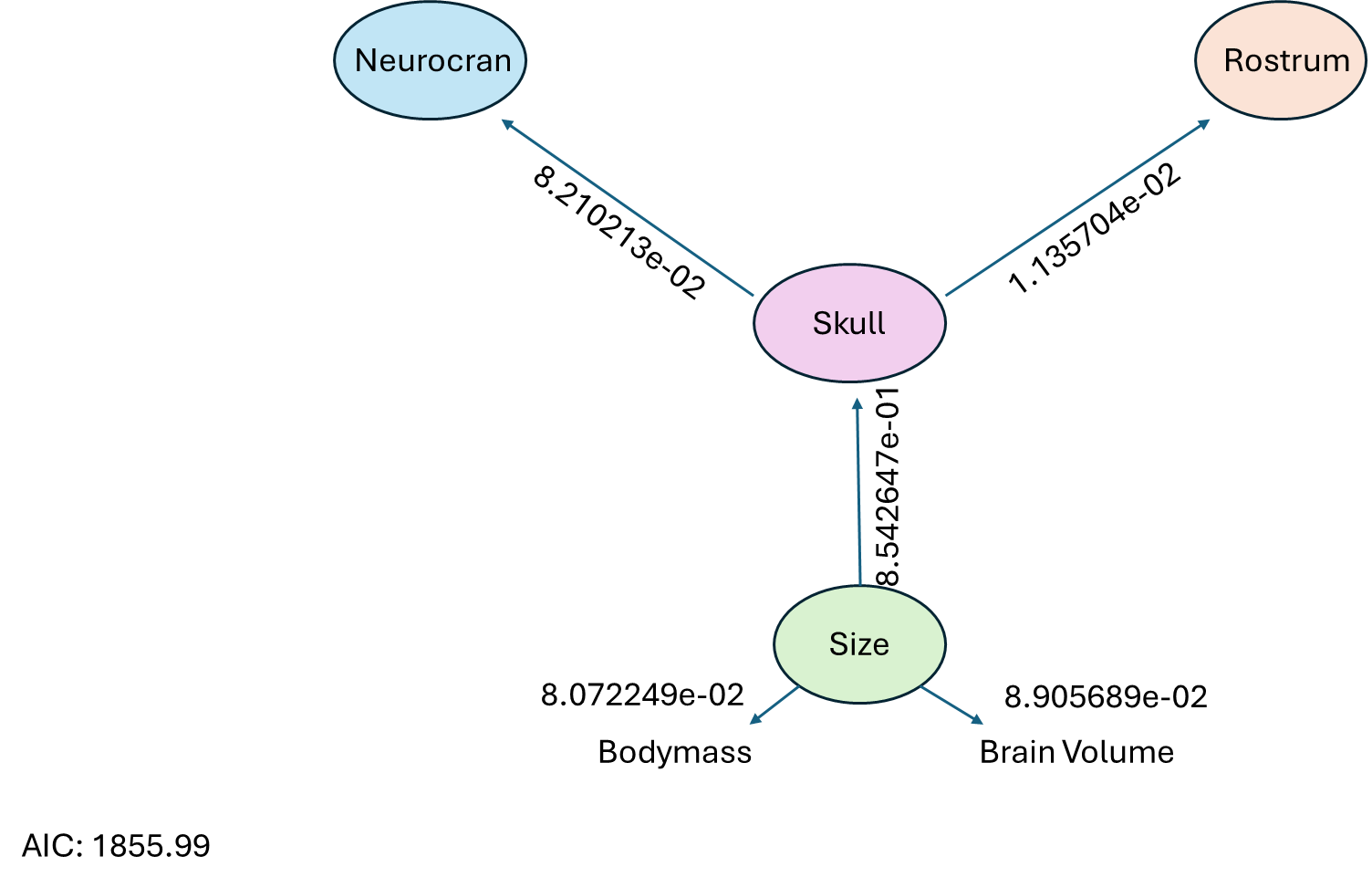
**

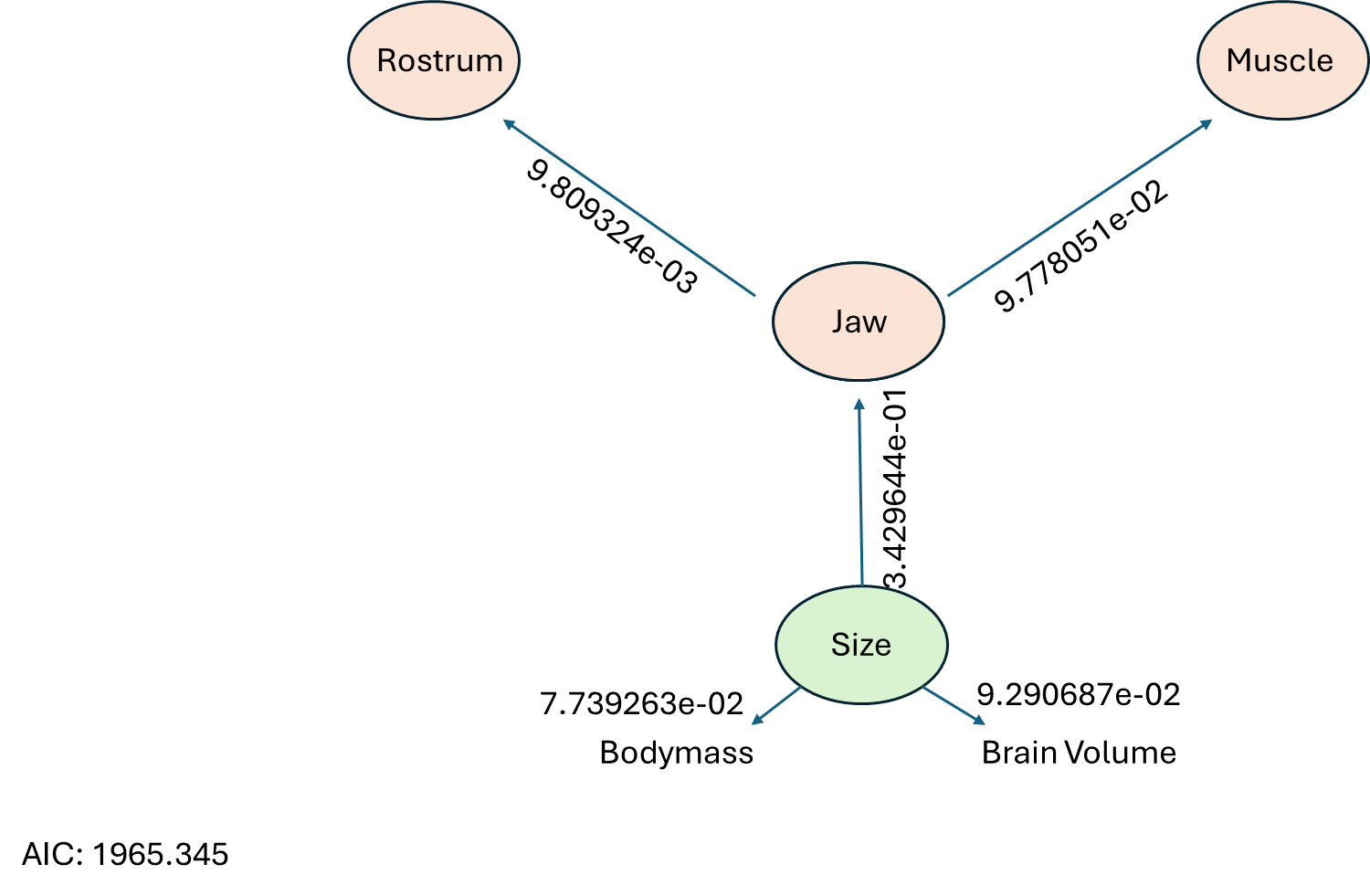


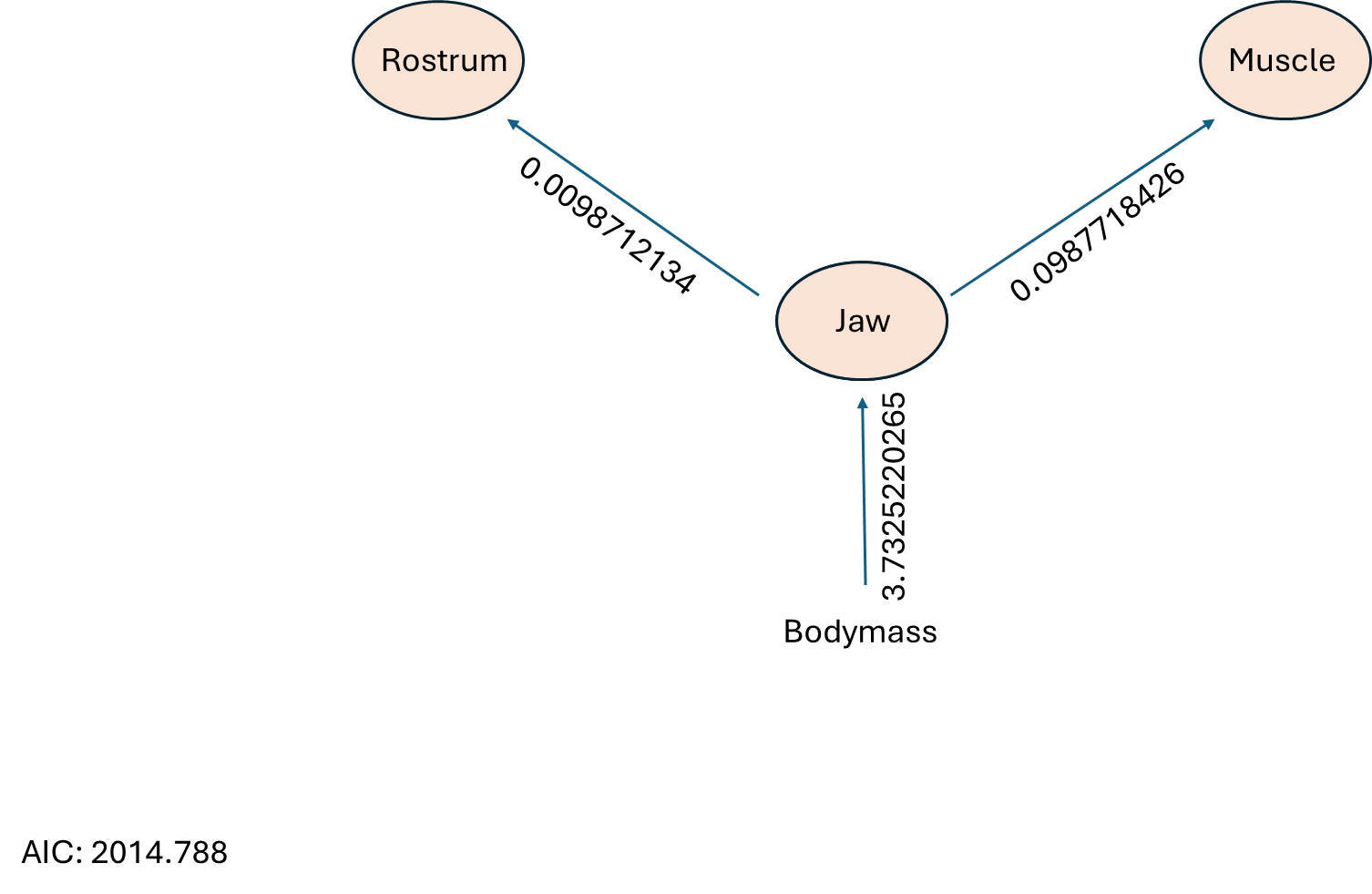


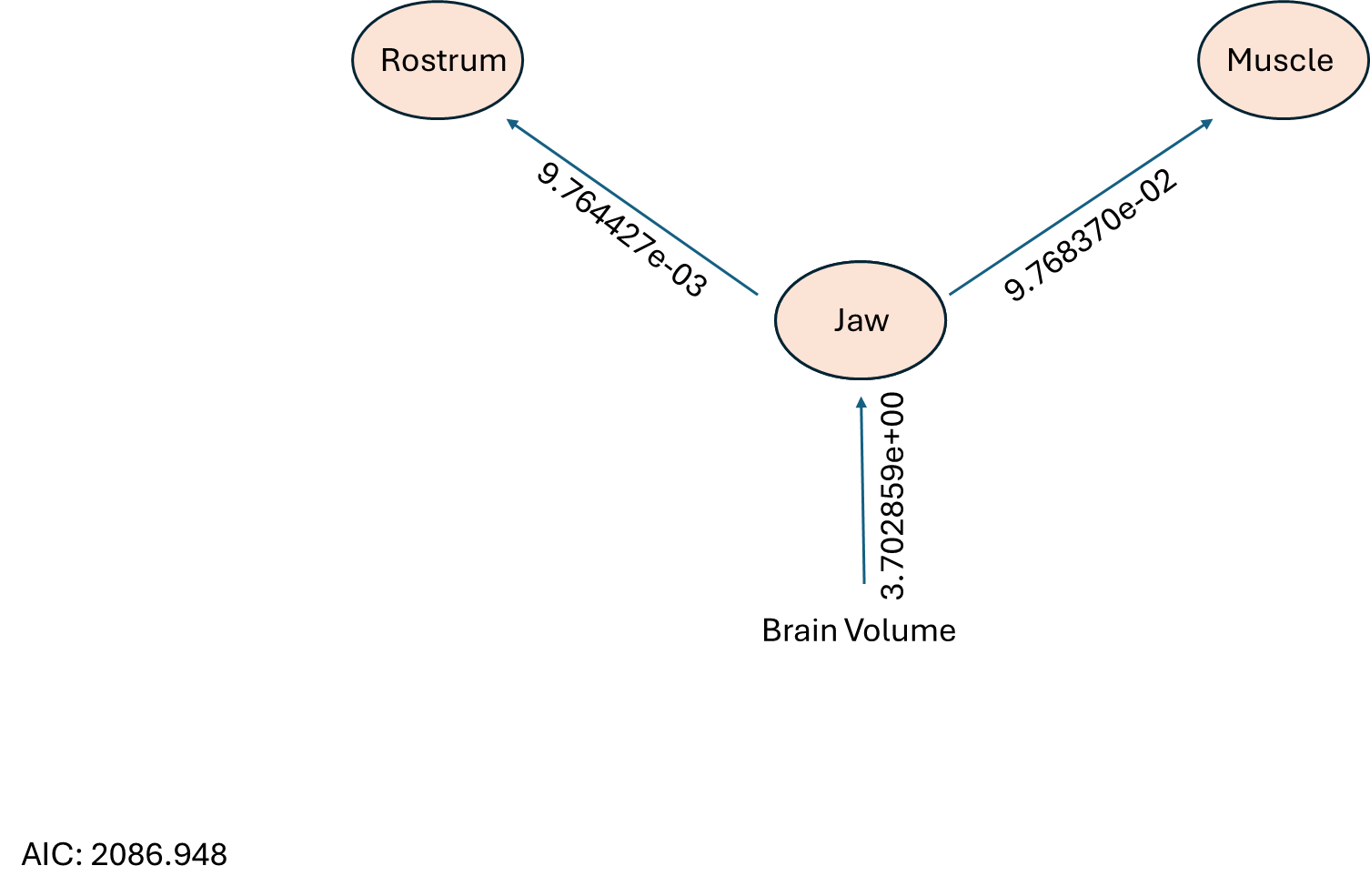


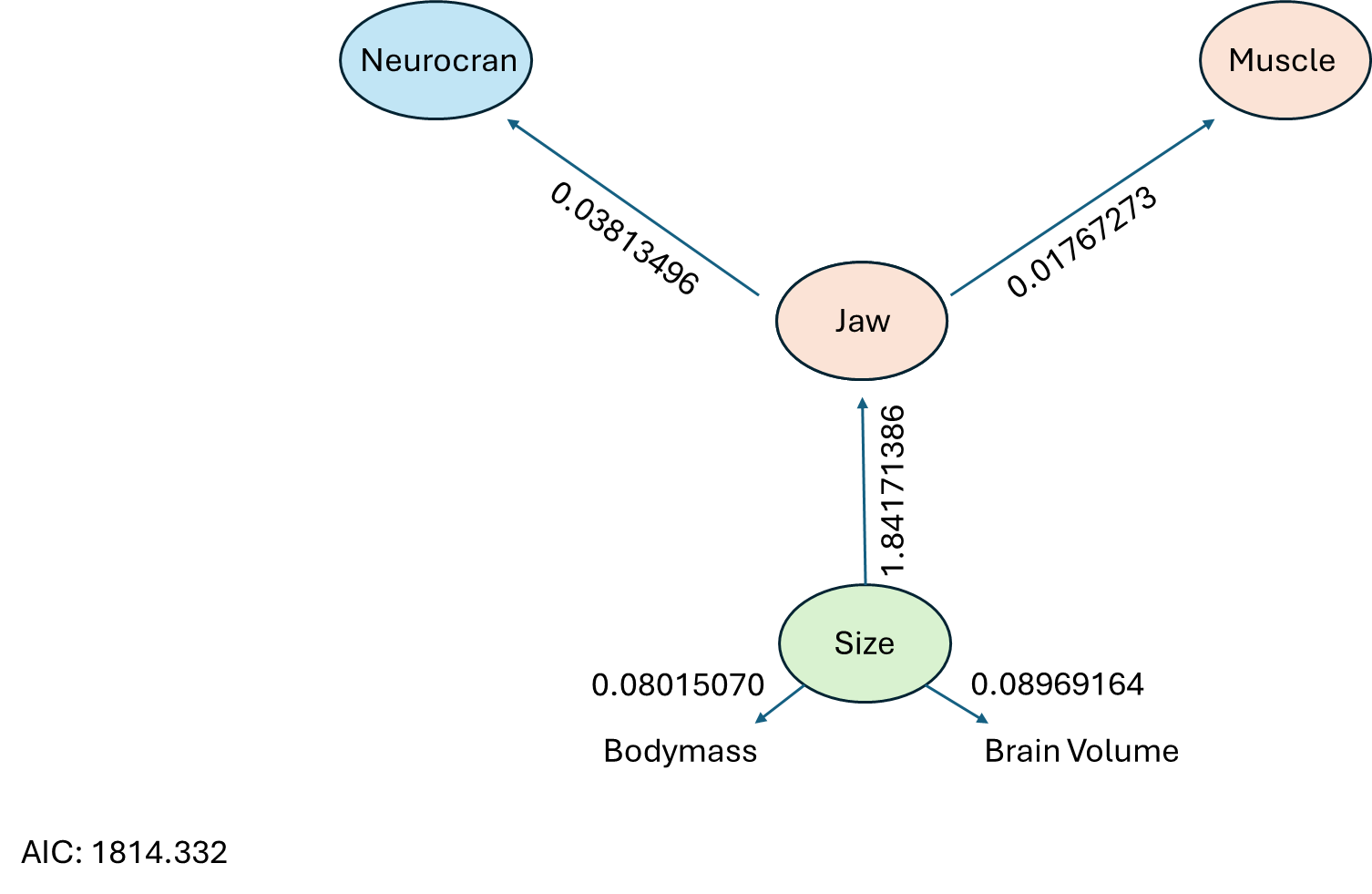


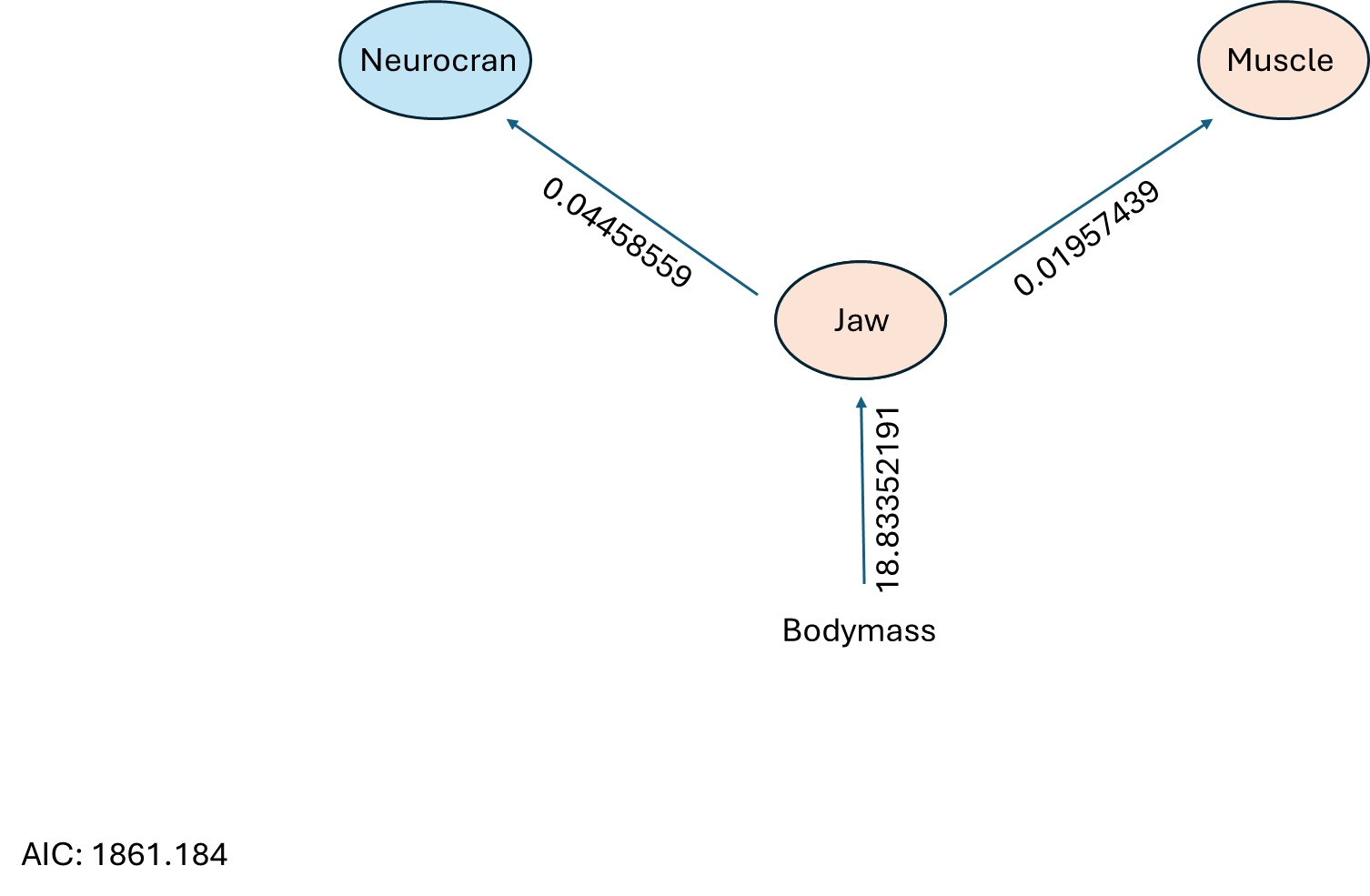


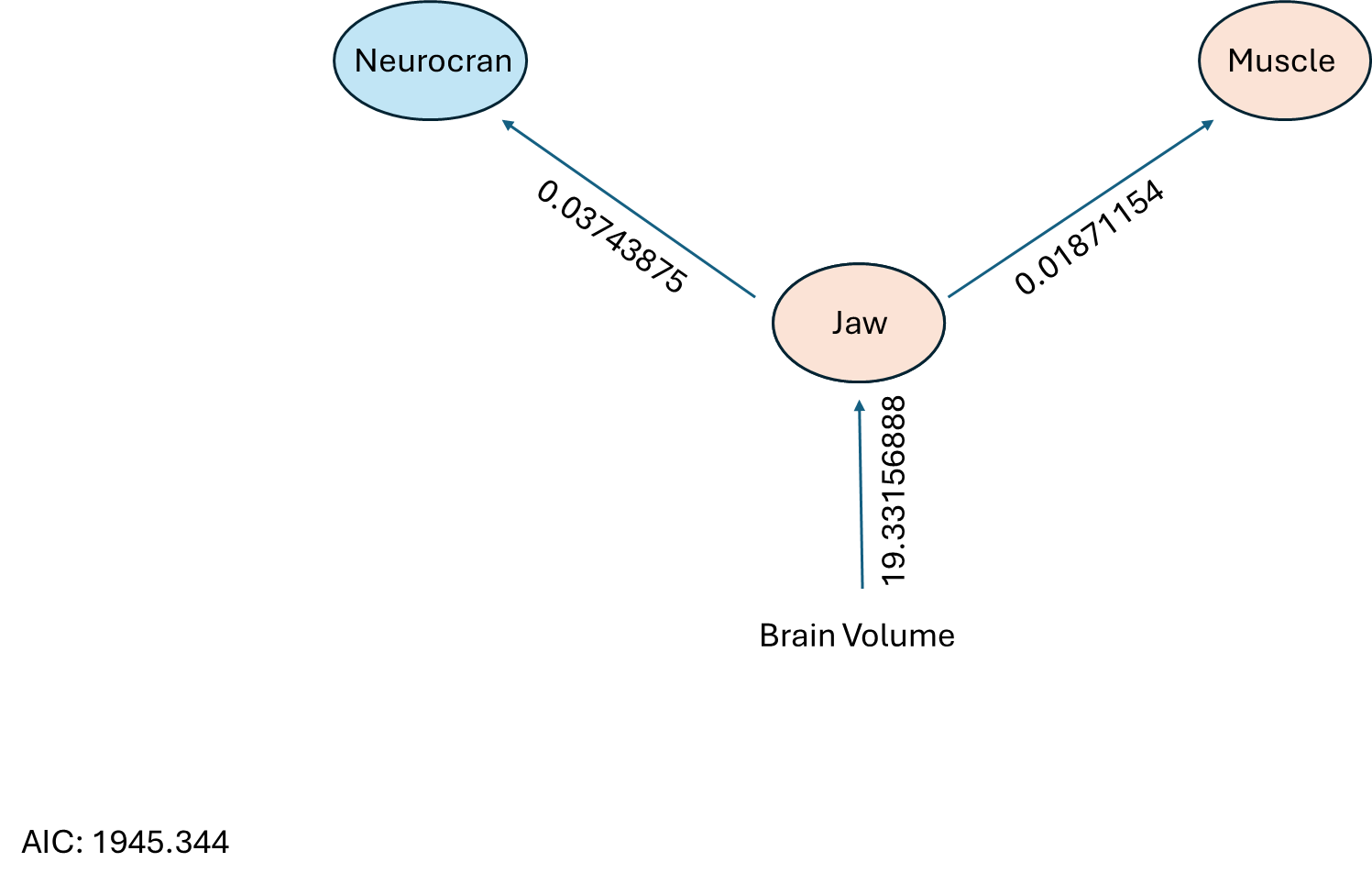


**
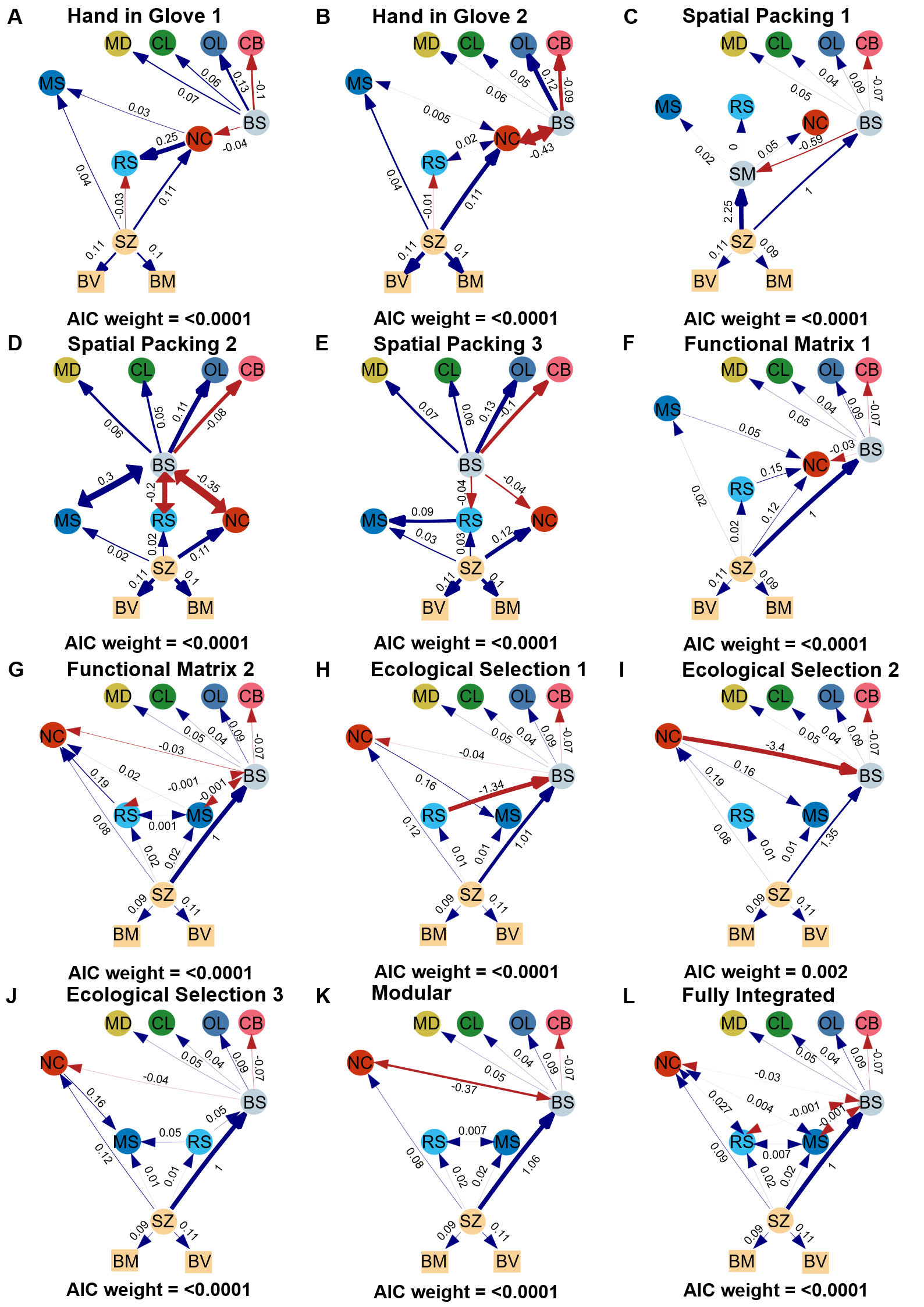
**

**Supplementary Figure 2: DAGs for SEMs Based on the Knapp et al. 2025 Models.** Colour coding and symbols match those in Figure 2 of the paper. Numbers represent path coefficients with the thickness of the arrows representing the strength of the covariances. Red arrows indicate negative correlations and blue arrows represent positive correlations. AIC weights represent the conditional probability that a given model has the greatest support in the set of models tested.

**
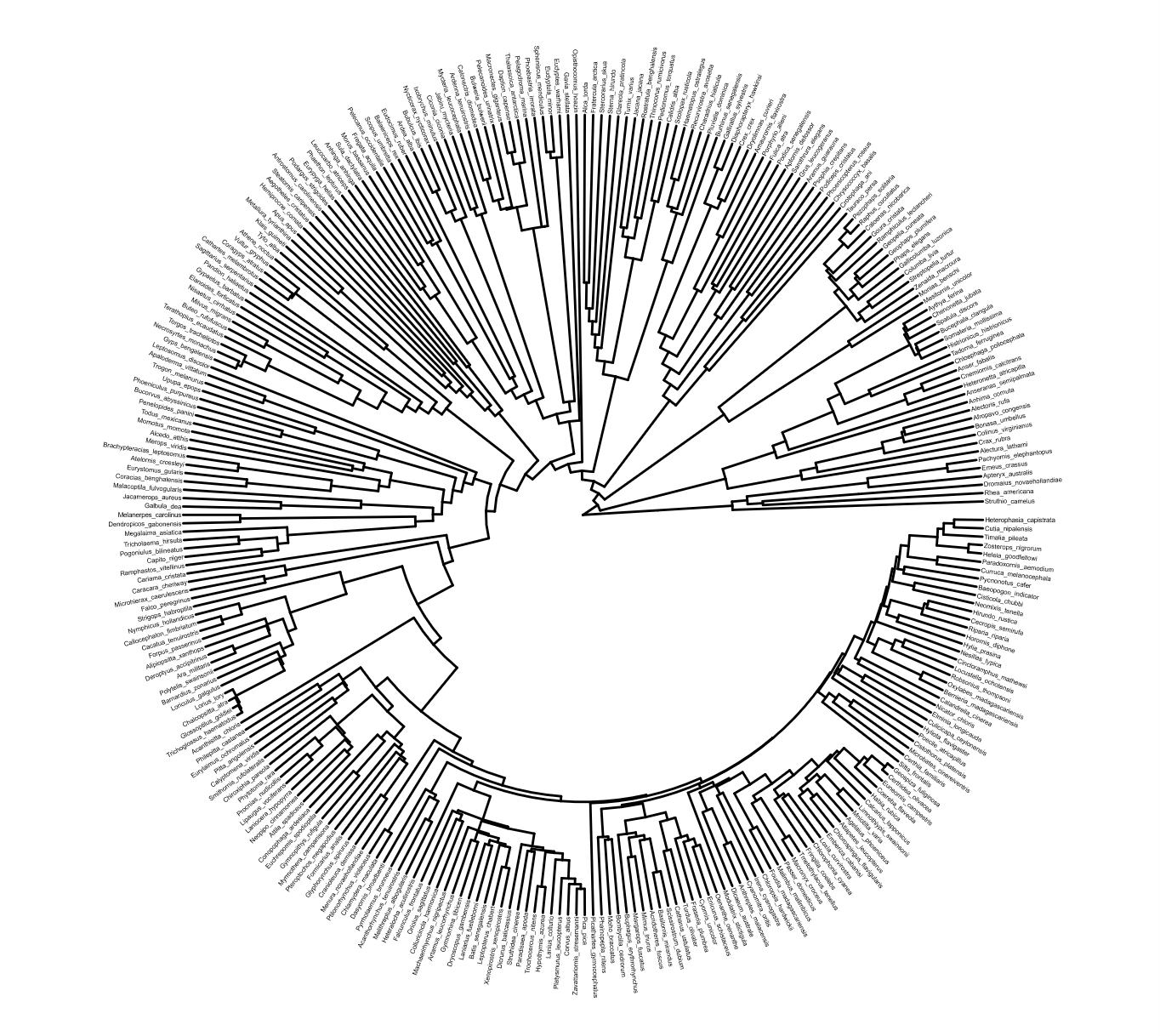
**

**Supplementary Figure 3a: Phylogeny Based on Claramunt et al. 2024**


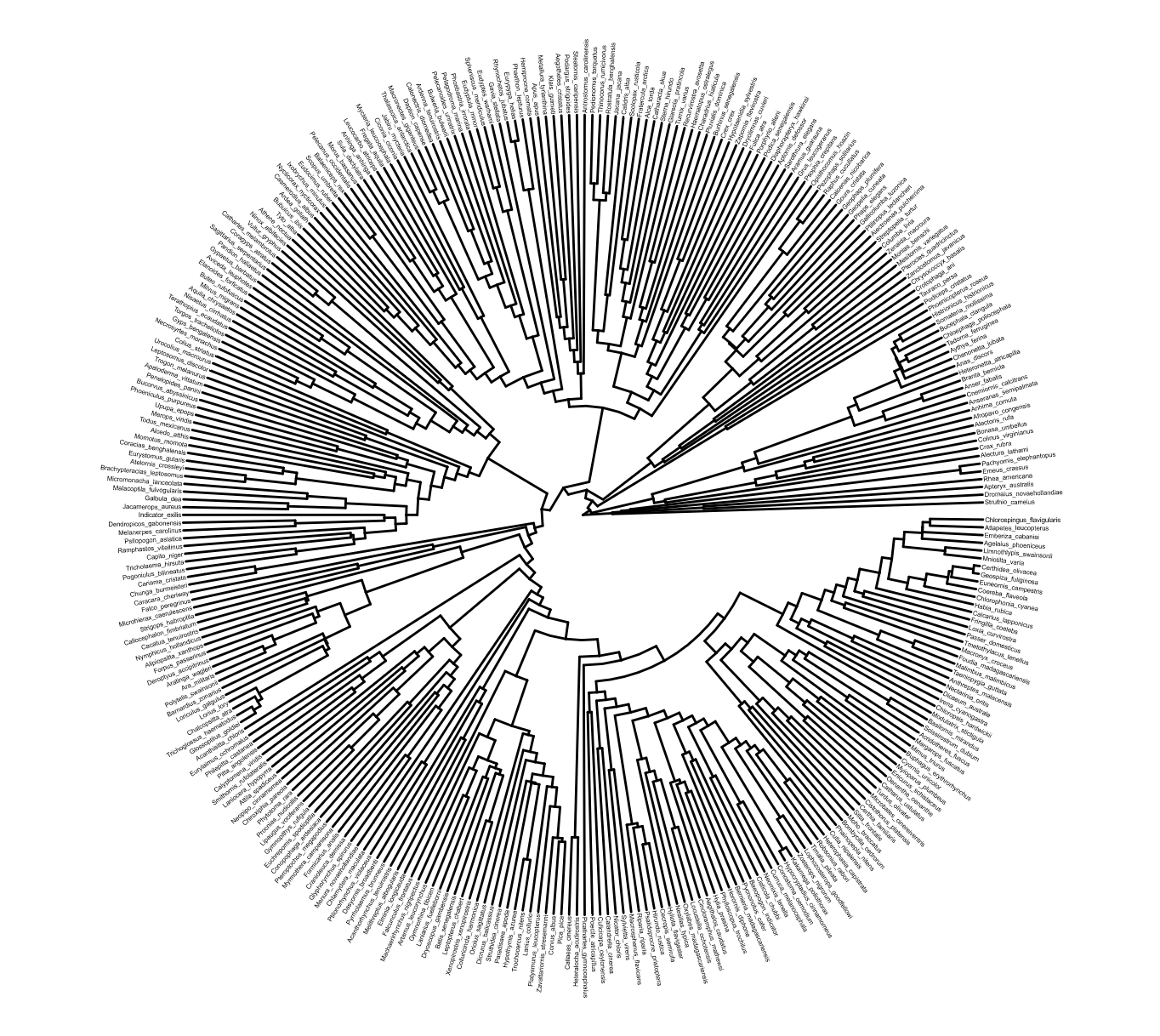


**Supplementary Figure 3b: Phylogeny Based on Stiller et al. 2024**


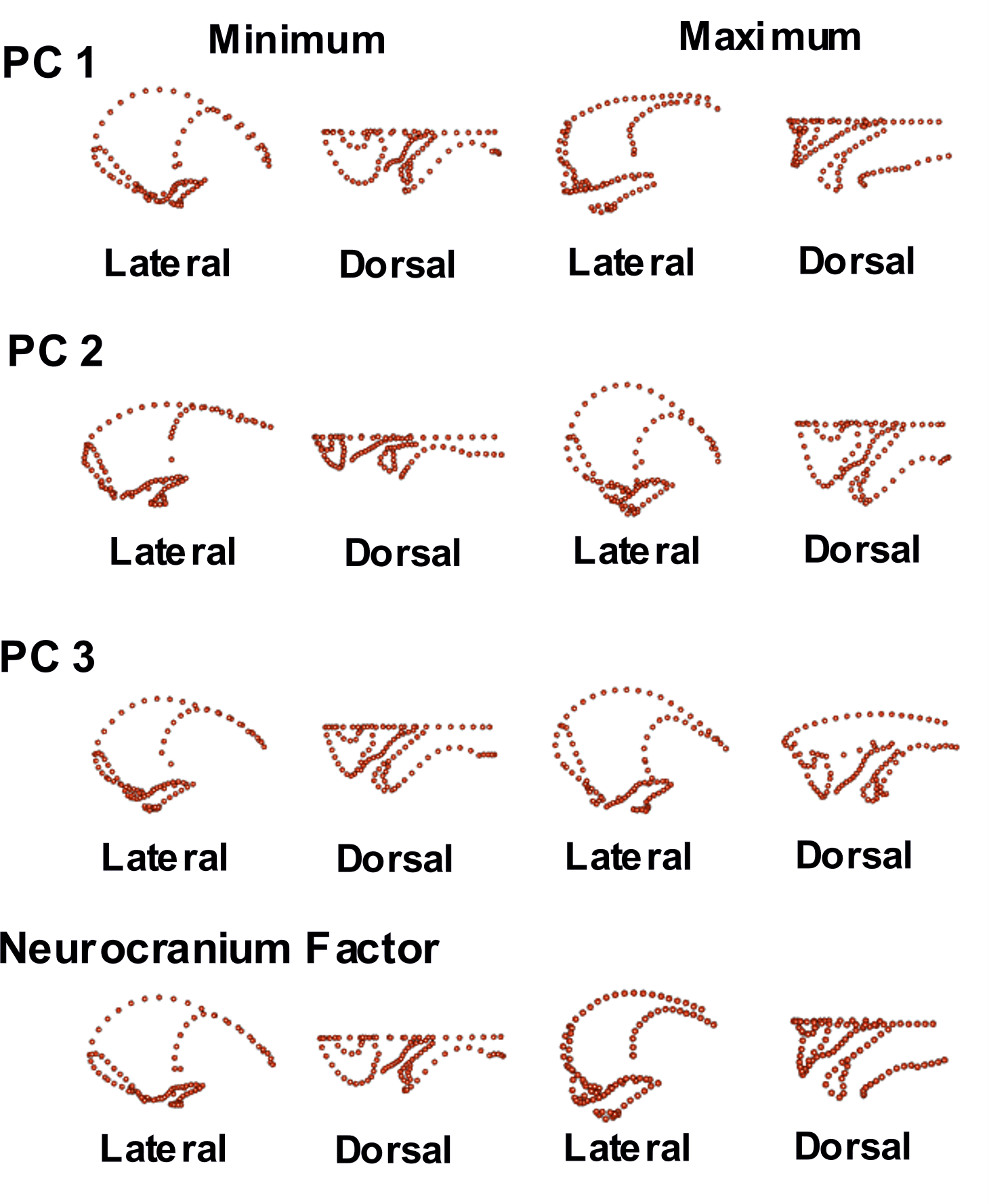


**Supplementary Figure 4:** Landmark configurations for minimum (left) and maximum (right) values of the first three principal components (PC 1, PC 2, PC 3) and the PFA (Neurocranium Factor) for the Neurocranium. Landmarks are taken from the right side of the skull and shown in lateral and dorsal views.


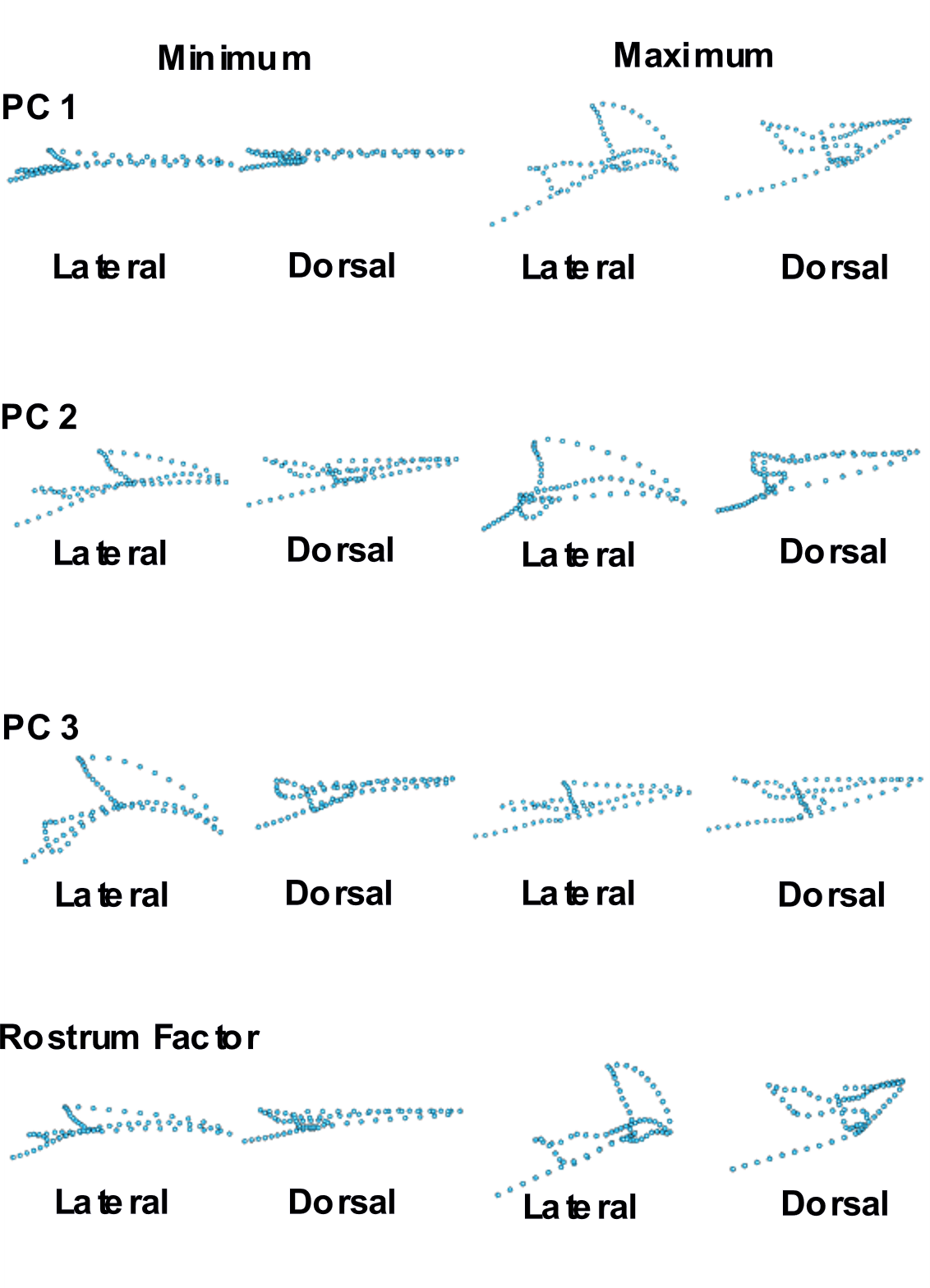
**Supplementary Figure 5:** Landmark configurations for minimum (left) and maximum (right) values of the first three principal components (PC 1, PC 2, PC 3) and the PFA (Rostrum Factor) for the Rostrum. Landmarks are taken from the right side of the skull and shown in lateral and dorsal views.


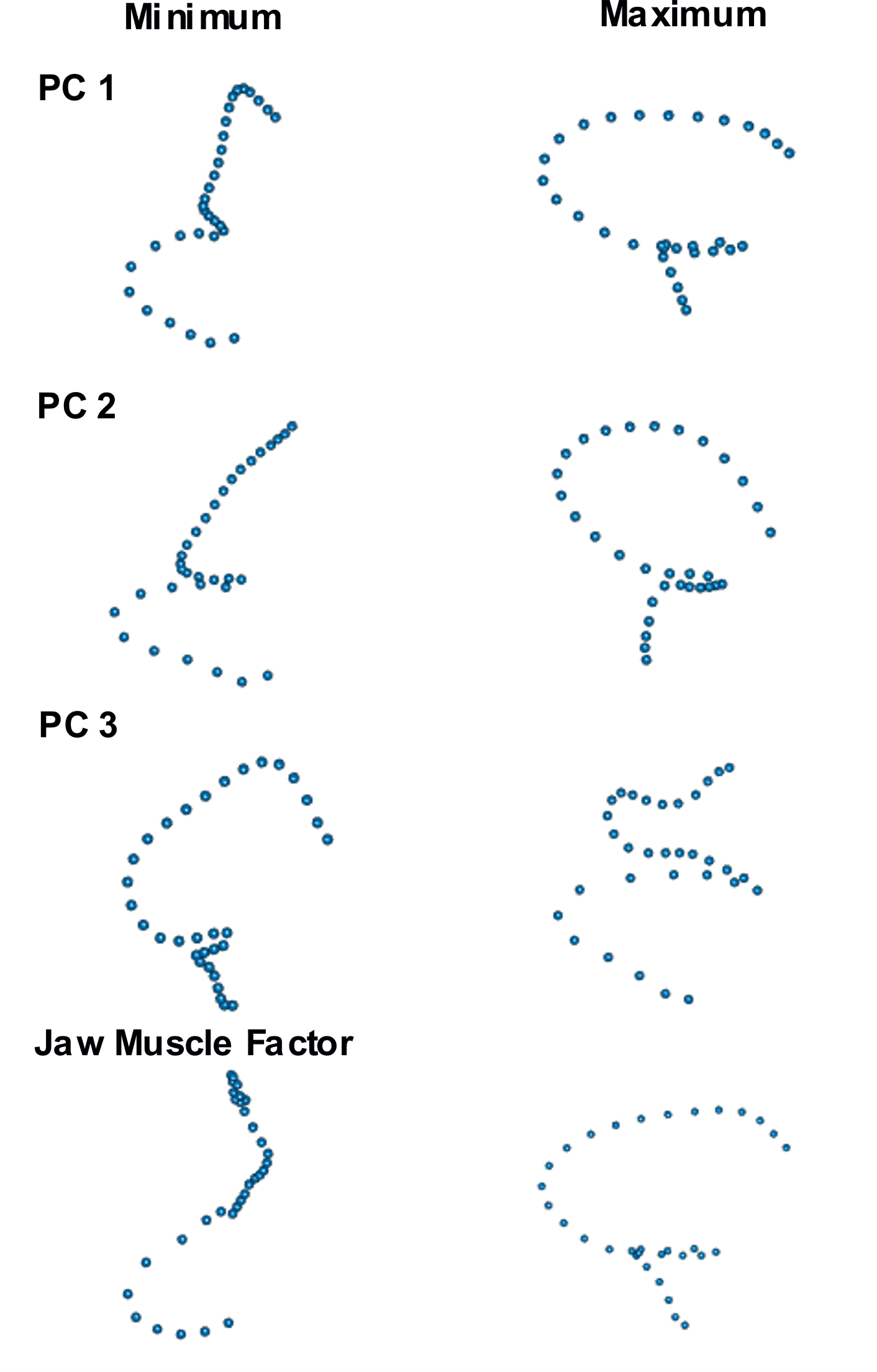


**Supplementary Figure 6:** Landmark configurations for minimum (left) and maximum (right) values of the first three principal components (PC 1, PC 2, PC 3) and the PFA (Jaw Muscle Factor) for the Jaw Muscle attachment sites. Landmarks are taken from the right side of the skull and shown in lateral and dorsal views.


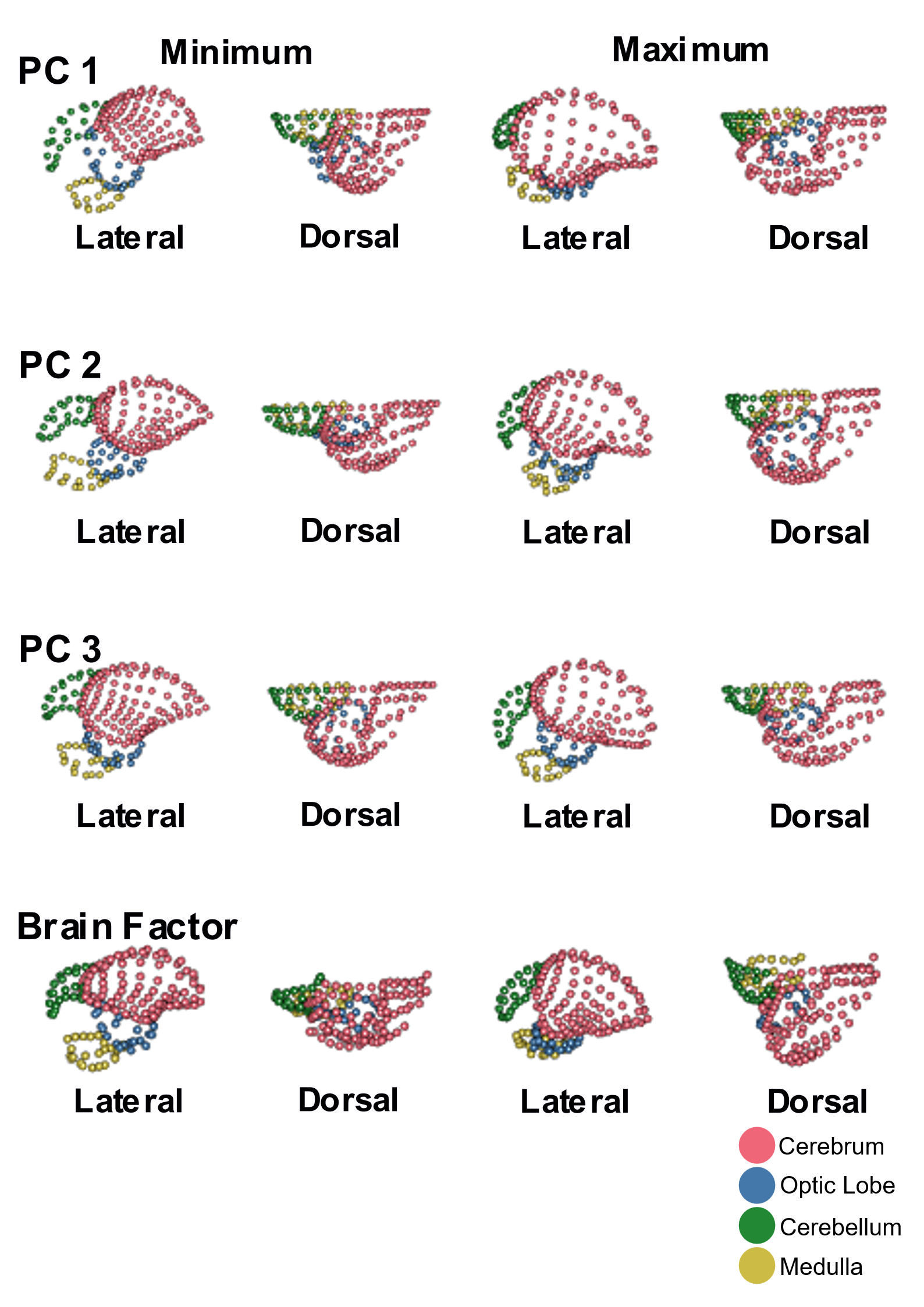


**Supplementary Figure 7:** Landmark configurations for minimum (left) and maximum (right) values of the first three principal components (PC 1, PC 2, PC 3) and the PFA (Brain Factor) for brain shape. Landmarks are taken from the right side of the endocast and shown in lateral and dorsal views.
